# System-level reorganization of gene networks underlies the nurse-to-forager transition in honeybees

**DOI:** 10.1073/pnas.2623939123

**Published:** 2026-10-22

**Authors:** Robert E. Page, Eric Bonabeau

**Affiliations:** ^a^ School of Complex Adaptive Systems, Arizona State University, Tempe, AZ 85281-2701

**Keywords:** behavioral genomics, *Apis mellifera*, network analysis, statistical physics

## Abstract

Division of labor in social insects is enabled by coordinated changes in physiology, neural signaling, and gene regulation, yet it remains unclear to what extent behavioral maturation reflects incremental changes in individual genes and/or a reorganization of transcriptomic regulation at the system level. Here, we analyze the nurse-to-forager transition in worker honeybees (*Apis mellifera*) from a network perspective, treating the transcriptome as a gene–gene correlation system whose global organization reflects collective regulatory states. Using fat body gene expression data across five developmental time points that span an interval of behavioral transition, we construct signed gene–gene correlation networks and quantify their macro- and mesostate properties. We then descend to the level of the gene and construct microstate gene lists of interacting genes. Behavioral maturation of worker honeybees is accompanied by coordinated, nonmonotonic changes in global network structure rather than simple global shifts in interaction strength across the network. Early stages exhibit broadly positive, globally synchronized correlations, whereas later stages show increased modular segregation and the emergence of antagonistic gene–gene relationships. Despite this differentiation, networks remain fully connected with high global efficiency, consistent with a flexible small-world–like organization. Positive correlations primarily support global integration, while negative correlations act as localized, boundary-enforcing constraints. Negative correlations form a structured pattern of antagonism that is sparse and module-specific, occurring primarily between functional gene modules rather than within them. Network layouts and module analyses reveal two polarized, attractor-like regulatory states, a structure recapitulated in a vitellogenin-centered subnetwork and consistent with prior experimental perturbation studies.

## Significance

Division of labor in social insects emerges from interactions among social partners and physiological and molecular processes. However, most gene network studies of behavior explain behavioral changes in terms of expression changes in individual genes or gene members of networks. By analyzing the nurse-to-forager transition in maturing honeybees as a gene–gene correlation network, we show that behavioral maturation corresponds to a system-level reorganization of gene network structure involving coordinated changes in global network properties, the emergence of antagonistically correlated gene modules, and the formation of distinct network macrostates. These results suggest that behavioral differentiation is associated with collective changes in gene regulatory dynamics, providing a systems-level framework for linking molecular organization to behavioral phenotypes.

Division of labor among adult worker honeybees (*Apis mellifera*) provides a tractable and well-characterized model for studying how complex behavioral states arise from interacting molecular and physiological processes. Worker honeybees undergo an age-related and socially mediated transition from in-nest tasks (e.g., brood care and food processing) to foraging outside the nest, typically during their second to fourth weeks of adult life (1, 2). This age-related polyethism is associated with robust shifts in gene-expression profiles and can be experimentally dissociated from chronological age through manipulation of the social environment (3–6).

Behavioral ontogeny coincides with coordinated changes across multiple physiological systems, including endocrine signaling (juvenile hormone and ecdysteroids), neuromodulatory pathways (biogenic amines such as octopamine, dopamine, and serotonin), metabolism (insulin/IGF signaling, vitellogenin, and hexamerins), and somatic physiology (hypopharyngeal gland development and ovary activation). Among these factors, juvenile hormone (JH), vitellogenin (VG), and nutrient-sensitive signaling cascades have been implicated as key regulators of the pace and expression of foraging behavior (2, 6, 7). Manipulative and correlative studies demonstrate that these molecular and physiological components interact to bias the timing of foraging onset and to shape foraging preferences and lifespan (2, 6, 8–10).

Although some sociogenomic studies have applied coexpression and systems-level approaches to behavioral maturation and social behavior (11–13), most network analyses of the nurse-to-forager transition have focused on identifying differentially expressed genes in candidate regulatory pathways, yielding valuable mechanistic hypotheses about individual genes and pathways underlying behavior (4, 8, 14, 15). While this gene-centric approach has been highly productive, it does not by itself resolve whether behavioral phenotypes arise from the additive effects of a limited number of key regulators, or from coordinated reorganization of the correlation architecture of many interacting components.

An alternative and complementary framework treats the transcriptome as a high-dimensional relational system, in which interactions among genes give rise to behavior-related gene regulatory networks and collective regulatory states (16). Gene coexpression and correlation network methods operationalize this perspective by summarizing pairwise covariation across individuals or conditions, thereby revealing functional modules and coordinated variation without requiring prior assumptions about regulatory directionality (17). In this view, correlation structure represents an intermediate level of organization linking underlying molecular regulatory processes to organismal phenotypes.

From the perspective of dynamical systems and statistical physics, regulatory networks composed of many interacting elements can exhibit qualitatively distinct macroscopic behavior, or macrostates, that emerge from underlying microstate interactions (18, 19). Empirical and theoretical work across cell biology supports the idea that phenotypic states—such as cell fates—can correspond to high-dimensional, attractor-like configurations in gene-expression space (20–22), with transitions between states accompanied by coordinated reorganization of global correlation structure. Related ideas concerning attractor dynamics, criticality, and collective organization have also been applied to neural systems and other forms of biological collective behavior (18, 19).

Here, we adopt this systems-level perspective to ask whether the nurse-to-forager transition in worker honeybees corresponds to a reorganization of the global correlative structure of the transcriptome. Rather than inferring directed regulatory interactions or emphasizing individual gene effects (23), we represent a targeted subset of the fat-body transcriptome as a gene–gene correlation matrix and analyze its large-scale statistical properties (11, 24). The fat body is the principal site of vitellogenin production. Vitellogenin participates with juvenile hormone and nutrient-sensitive signaling pathways that regulate the nurse-to-forager transition (2, 6, 24). Nurses are generally characterized by a vitellogenin-rich physiological state, whereas the transition toward foraging involves coordinated changes in vitellogenin, juvenile hormone, metabolism, and associated signaling pathways (2, 11). The nurse-to-forager transition is not synchronous: As a cohort ages, some workers begin foraging while others of the same age remain engaged in nest tasks. Thus, the developmental interval examined here represents a population-level transition from a predominantly nurse-like state toward a mixture of nurse-like and forager-like workers, rather than the uniform conversion of all individuals from one behavioral state to another.

To characterize network organization at the macrostate level, we examine multiple complementary global measures of the gene–gene correlation network, including modularity, algebraic connectivity, mean signed and absolute correlations, and adjusted mutual information (AMI) (25–29). Together, these measures capture distinct aspects of global integration, modular differentiation, and polarization of gene regulatory relationships and provide a framework for assessing system-level organization (28). Using this framework, we show that behavioral maturation corresponds to a coordinated, system-level reorganization of gene network structure.

Throughout this study, we use terms such as “regulatory” and “network dynamics” descriptively to refer to patterns of functional coordination inferred from gene–gene correlation structure, without implying direct regulatory interactions or causal directionality.

## Results

### Data.

We analyzed gene expression data for 90 target genes plus vitellogenin (VG) titers in 80 newly emerged honeybee workers (*A. mellifera* L.), with 16 bees collected at 5 different ages (1, 3, 6, 10, and 15 d) (1, 2). This represents a population sample of gene and protein expression throughout the period of nurse bee to forager transition, a unique dataset that allows statistical analyses. Because gene–gene correlations are defined statistically across individuals, characterization of gene network structure requires sampling populations of individuals within a given context. Accordingly, network “dynamics” in this study refer to changes in ensemble correlation structure across age-defined populations rather than temporal trajectories within single individuals.

### The Network.

The gene–gene correlation network contained a total of 4,095 coefficients. Our operational gene–gene correlation network is defined as the set of statistically significant pairwise Spearman correlations $\rho_{s,ij}$ between genes i and j, with statistical significance of each association first assessed using the two-tailed *P*-value and then Benjamini–Hochberg (BH) corrected q < 0.05. The sign of the correlation determines whether the edge is positive ($\rho_{s,ij}>0$) or negative ($\rho_{s,ij}<0$), yielding a signed, weighted network $G=\left(V,E\right)$ for each day, where each edge $e_{\mathrm{ij}}\in E$ carries weight $w_{\mathrm{ij}}=\rho_{s,ij}\in \left[-1,1\right]$ and ${\text{sign}}_{\mathrm{ij}}=\text{sgn}\left(\rho_{s,ij}\right)\in \left\{-1,+1\right\}$.

### Macrostate-Level Organization of the Gene Network.

**Fig. 1.**
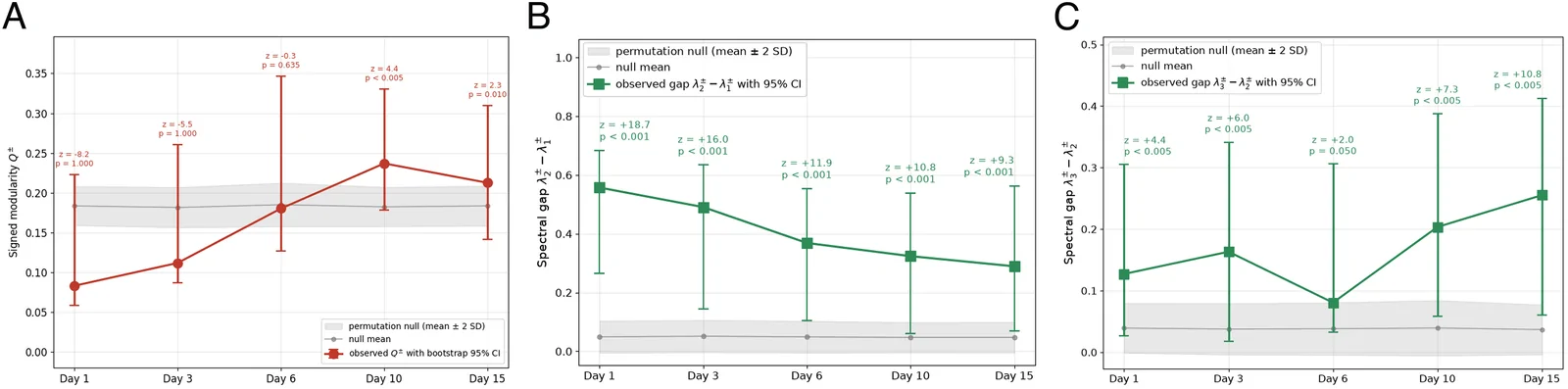
(*A*–*C*) (*A*) Observed signed modularity (Q±) with bootstrap 95% CI and permutation-null mean ±2 SD (gray band). Significant modular organization emerges at Days 10 and 15. (*B*) Spectral gap λ_2_± − λ_1_± is consistently above the null model. (*C*) Spectral gap (λ_3_± − λ_2_±), which reaches its minimum at Day 6 and maximum at Day 15.

To characterize global network organization, we examined signed modularity and the signed Laplacian spectrum (Fig. 1). Signed modularity measures partitioning into coordinated gene modules, whereas the Laplacian‘s first eigenvalue and the spectral gap characterize the emergence of bipolar organization.

Signed modularity was not greater than the permutation null on Days 1, 3, or 6 (z = −8.2, −5.5, and −0.3, respectively). Significant modular organization emerged at Day 10 (Q± = 0.24, z = 4.42, *P* < 0.005) and persisted at Day 15 (Q± = 0.21, z = 2.31, *P* = 0.01); bootstrap 95% CI exceeded the null mean only at these two ages (Fig. 1*A*).

#### Signed Laplacian analysis.

The signed Laplacian spectrum provides a complementary view of this reorganization (Fig. 1 *B* and *C*). The smallest eigenvalue λ_1_±, an indicator of frustration, is consistently significantly lower than the null model (z = –16 to –18, *P* < 0.001) and close to 0 (*SI Appendix*, Fig. S7), indicating a tendency to organize into balanced gene modules, whereas the spectral gap (λ_2_± − λ_1_±) measures how clearly one bipolar partition is distinguished from alternatives. It is consistently significantly higher than the null model (z = +9 to +16 *P* < 0.001), suggesting that the network possesses a clean 2-module split (Fig. 1*B*).

#### Structural balance analysis.

Granular structural balance provides an additional view of the network reorganization. Although the fraction of negative correlations remains low early in development and increases sharply at Day 10 (*SI Appendix*, Fig. S1), the network remains perfectly structurally balanced at all ages. A balance of 1 was observed on all days—a measure of the ratio of balanced (+/+/+ and +/-/-) to unbalanced (-/+/+ and -/-/-) triangles. During Days 1 to 6, this balance arises because correlations are overwhelmingly positive. By Days 10 and 15, positive correlations occur primarily within gene modules and negative correlations primarily between them, consistent with the emergence of two internally coherent, antagonistically related gene groups.

#### Spectral gap $\lambda_{3}^{\pm }-\lambda_{2}^{\pm }$.

The spectral gap (Fig. 1*C*) provides another view. The gap is substantially above null at every age, but its trajectory is nonmonotonic: large early, smallest at Day 6, then rising to its largest value at Day 15 (z=+10.8, the strongest single z-score). This highlights “Day 6 reorganization” and the emergence of a bipolar sub-network. By Day 15, the gap has more than tripled relative to Day 6, indicating that the network accommodates other genes that are not in either module, particularly on Days 10 and 15 when it reaches its maximum value. At Day 15, the network exhibits both a low λ_1_± (λ_1_± = 0.14, z = –16.1 vs. permutation null), a large first spectral gap (λ_2_± − λ_1_± = 0.29, z = +9.3) as well as a large second spectral gap (λ_3_± − λ_2_± = 0.26, z = +10.8) indicating consolidation of a structurally near-balanced architecture with two antagonistic gene modules and additional genes that don‘t belong in either module.

### Changes in Correlative Structure and Emergence of Antagonism.

To determine whether network reorganization reflects changes in correlation strength or sign, we examined mean absolute and signed correlations across development (*SI Appendix*, Fig. S1). Neither mean absolute correlation (*P* = 0.285) nor mean signed correlation (*P* = 0.505) changed significantly, whereas the fraction of negative correlations increased significantly. Thus, developmental reorganization primarily involves redistribution of correlation signs rather than changes in overall correlation magnitude, producing increasingly antagonistic organization while preserving overall coupling strength.

### Mesostate-Level Structure.

#### Force-directed layout.

**Fig. 2.**
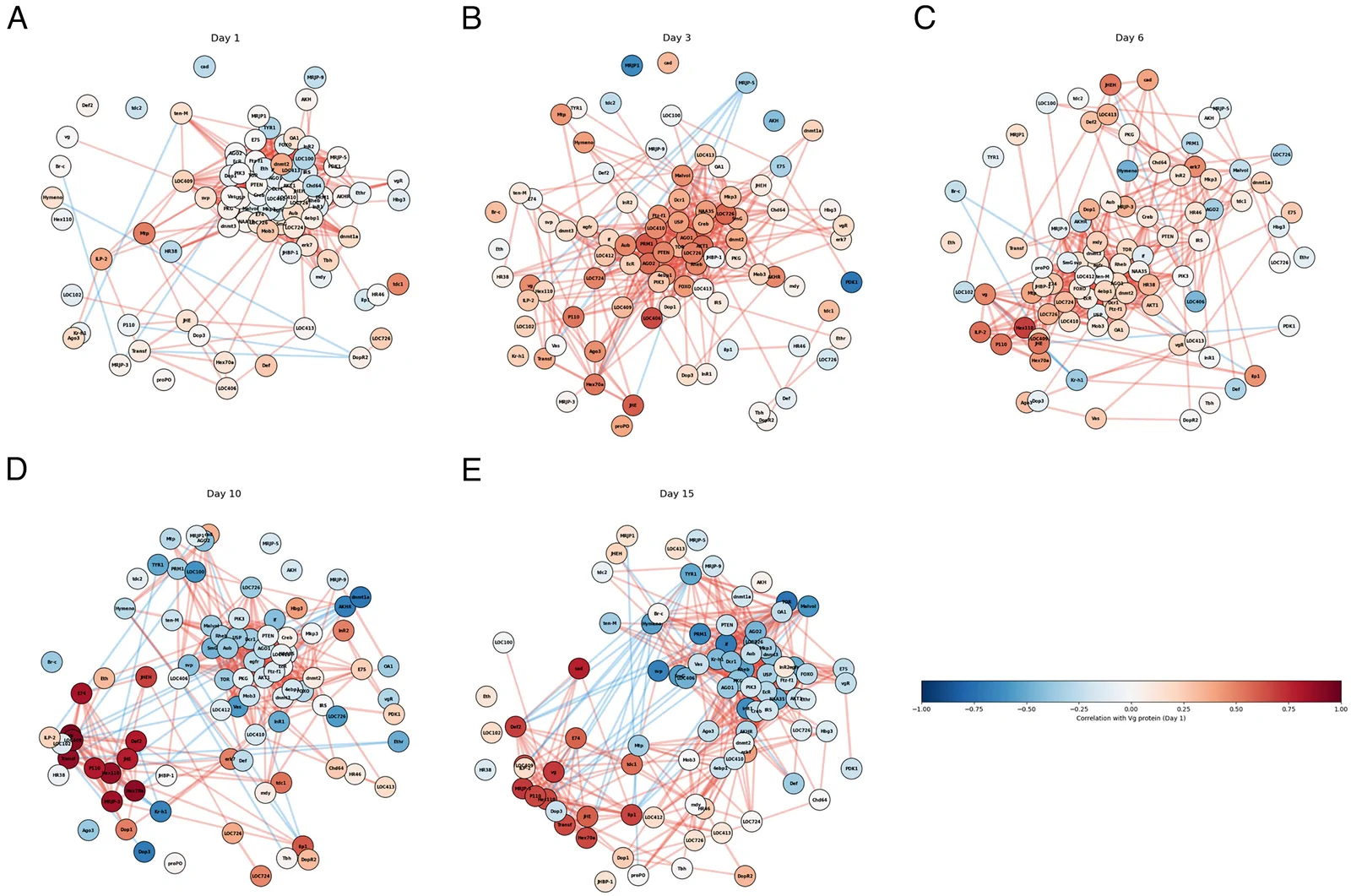
Force-directed layout [NetworkX spring_layout (30) with k = 0.7, 50 iterations] of the full correlation networks on Days 1, 3, 6, 10, and 15 (*A*–*E*). All nodes are present, even when the BH-corrected q-value ≥ 0.05. Node colors represent the corresponding gene expressions’ correlations with VG titers (red = positive, blue = negative, q not considered). Edges are characterized by thickness and color, with thickness representing the absolute value of the correlation and color indicating its sign; only q < 0.05 correlations are used as edges.

Force-directed layouts provide a visual representation of the network reorganization identified by the preceding quantitative analyses (Fig. 2). The transition is apparent by Day 6, followed by the emergence of two dominant, antagonistically related clusters on Days 10 and 15.

#### Vitellogenin.

Consistent with previous studies, VG titers increased during early adult life, reached a plateau by Day 6, and remained elevated through Days 10 and 15 (*SI Appendix*, Fig. S2) (2, 31–34). This confirms that the sampled bees span the critical developmental interval during which some workers are expected to transition toward foraging whereas others remain nurse-like.

VG titers differed significantly among ages (Kruskal–Wallis H = 22.5, *P* = 1.6 × 10^−4^) and exhibited a significant monotonic increase with age (Spearman ρ = +0.48, *P* = 6.9 × 10^−6^). Post hoc Dunn’s tests (BH corrected) showed that Days 6, 10, and 15 did not differ significantly from one another (q ≥ 0.66), whereas all three were significantly higher than Day 1 (q ≤ 0.005) and Day 3 (q < 0.05) (*SI Appendix*, Fig. S2).

Variation in VG also increased across development (Levene’s test W = 4.05, *P* = $5.0\times 10^{-3}$), with the SD increasing approximately fourfold between Days 1 and 10 and the robust quartile coefficient of dispersion (QCD) reaching its maximum at Day 15 (0.99 vs. 0.24 on Day 1). Gaussian mixture analysis of $\log\left(1+VG\right)$) provided no evidence for bimodality at either Day 10 $\left(\Delta \text{BIC}=-8.3\right)$ or Day 15 $\left(\Delta \text{BIC}=-0.7\right)$, indicating that the increased dispersion reflects a continuous broadening of physiological states rather than two discrete VG-defined worker subpopulations.

#### Consensus clustering.

Consensus clustering was applied to each day’s network. The number K of clusters identified on each day varies and so does cluster stability. K = 4 on Days 1, 10, and 15; K = 3 on Days 3 and 6. Examining the clusters at Day 15, we find the following statistically robust gene “modules” with clear functional interpretation (Table 1). One important observation is that Modules M1 and M2 are characterized by two properties: 1) strong correlations with VG titers and 2) strong positive within-module correlations and strong negative correlations between modules. The other two modules have weak or nonsignificant correlations with VG protein titers and weak correlations within or between modules. We infer from this observation that modules M1 and M2, which we will from now on call H (for high VG titers) and L (for low VG titers), drive the evolution of the network structure. Module H (13 genes) represents the nurse-associated program, Module L (14 genes) the forager-associated program. The remaining 63 genes form a more “neutral” set with respect to VG dynamics.

**Table 1.** List of genes in the 4 modules detected from consensus clustering and functional interpretation. Functional modules were defined based on shared biological roles and conserved signaling pathways identified from Gene Ontology annotations, pathway databases, and published functional studies in honeybees and other insects.

Module	n	Members	Mean ρ(s, VG)	Theme	Functional rationale
M1	13	vg, Hex70a, Hex110, Transferrin1, ilp1, P110, Def2, JHE, LOC409966, MRJP-3, cad, E74, tdc1	+0.66	Pro-VG/nurse/JH-inactivation	Yolk-protein machinery (vg, hexamerins, transferrin) cocluster with the brood-food MRJP-3, the nurse-biased insulin ligand ilp1, the upstream PI3K subunit P110, and three independent JH-inactivating mechanisms (JHE, JHEH, JHBP-1). Def2 reflects VG’s documented immune-protective role in workers. The composite signature is the canonical nurse fat-body program.
M2	14	Malvolio, PRM1, TOR, Hymenoptaecin, SmG, LOC406144, svp, InR1, Def, if, Rheb, TYR1, AGO2, NAA35	−0.56	Anti-VG/TOReffector/forager	The TOR/Rheb effector axis of insulin signaling, paired with InR1 (antagonistic to the M1-resident InR2 by long-standing hypothesis), the canonical forager marker Malvolio, the tyramine receptor TYR1, forager state AMPs (Hymenoptaecin, Def), and the nuclear receptor svp. Module stability of 0.84 makes this the most robustly identified cluster—these genes cocluster in 84% of bootstrap × method runs.
M3	37	Mkp3, USP, EcR, PKG, Aub, AGO1, PIK3, Ftz-f1, dnmt3, Dcr1, PTEN, AKT1, IRS, Kr-h1, Vas, Creb, E75, Ethr, dnmt1a, LOC726899 GB52437, vgR, OA1, ten-M, LOC726648, Br-c, tdc2, Ago3, MRJP-9, PDK1, InR2, MRJP-5, AKH, Dop3, LOC100577942, Tbh, Eth, egfr	−0.22	VG-orthogonal regulatory machinery	The entire small-RNA + DNA-methylation machinery (AGO1, AGO2, Ago3, Aub, Dcr1, dnmt1a, dnmt3-7 genes coclustering as a unit), most of the EcR/USP ecdysone-receptor cassette, the upstream/proximal IIS arm (AKT1, IRS, PIK3, PTEN, PDK1), and the forager markers Kr-h1 and PKG.
M4	26	Mob3, mdy, LOC726781, dnmt2, proPO, LOC412541, LOC410022, Mtp, 4ebp1, ILP-2, AKHR, LOC724829, LOC413141, FOXO, Dop1, LOC413670, HR46, DopR2, LOC102655054, erk7, JHEH, HR38, Hbg3, Chd64, JHBP-1, MRJP1	+0.04	FOXO/lipid mobilization/dopaminergic	The classic low-IIS/catabolic fingerprint: FOXO with its direct target 4ebp1, the AKH receptor (glucagon analog driving energy mobilization), and lipid metabolism genes (Mtp, mdy). ILP-2 sits here apart from ilp1 (M1), and the dopamine arm (Dop1, DopR2) clusters apart from the tyramine arm (TYR1 in M2). Member-level stabilities are uniform but signed VG correlations cancel, so the module is coherent in coexpression but VG-orthogonal in mean.

M1 = H module, M2 = L module. Module functions are based Gene Ontology annotations, pathway databases, and published functional studies in honeybees and other insects.

### Adjusted Mutual Information.

**Fig. 3.**
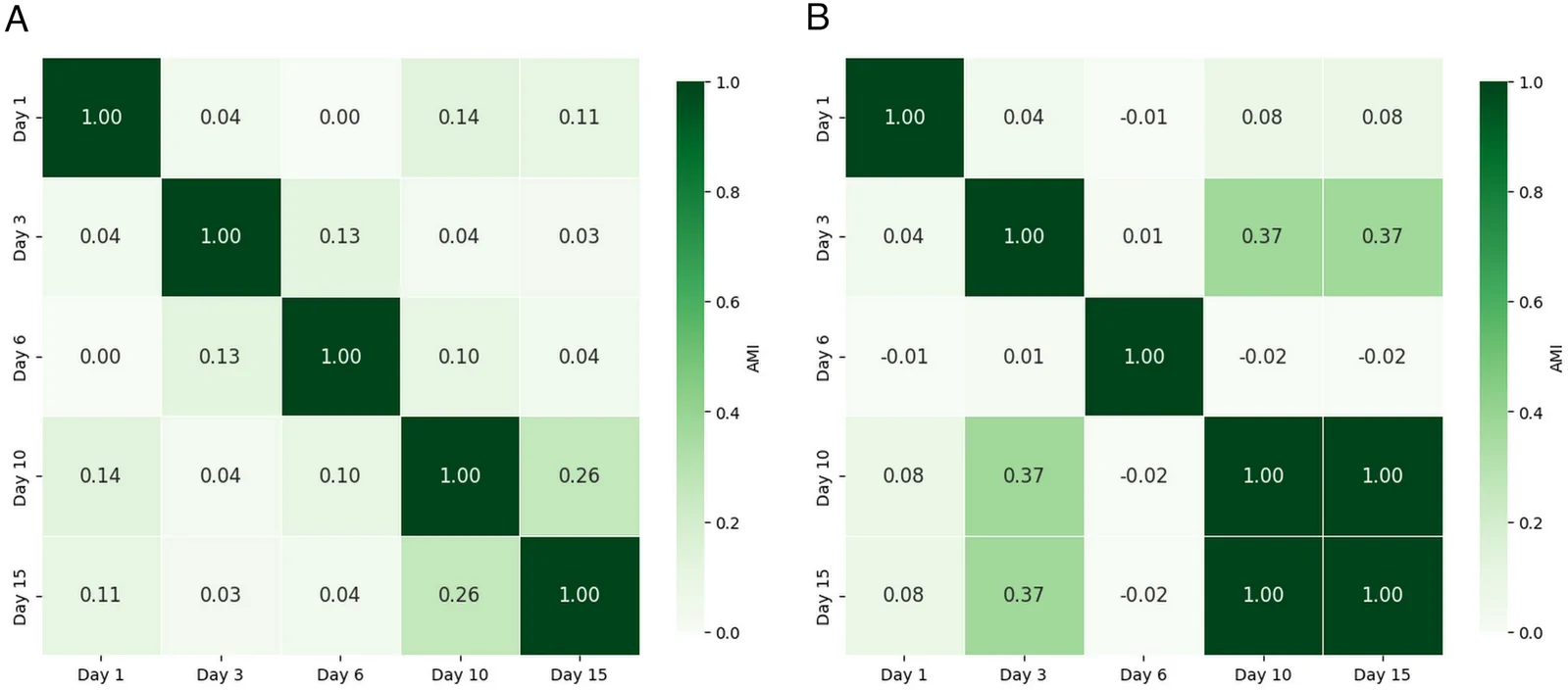
AMI between modules detected on Days 1, 3, 6, 10, and 15. (*A*) All genes. (*B*) The 27 genes comprising the Day 15 H and L modules. Module similarity is greatest between Days 10 and 15.

To determine how module membership changes across development, we quantified the similarity of community partitions between ages using Adjusted Mutual Information (AMI). AMI is a chance-corrected measure of overlap between two partitions. By construction, $E\left[AMI\right]=0$ for two independent random partitions and AMI=1 when the two partitions are identical. Module membership is highly dynamic across Days 1 to 6, followed by a major reorganization between Days 6 and 10 (Fig. 3*A*). In contrast, the higher similarity between Days 10 and 15 indicates stabilization of module membership after this transition. This pattern is particularly pronounced for the 27 genes comprising the Day 15 H and L modules, whose organization is largely established by Day 10 and retained through Day 15 (Fig. 3*B*).

**Fig. 4.**
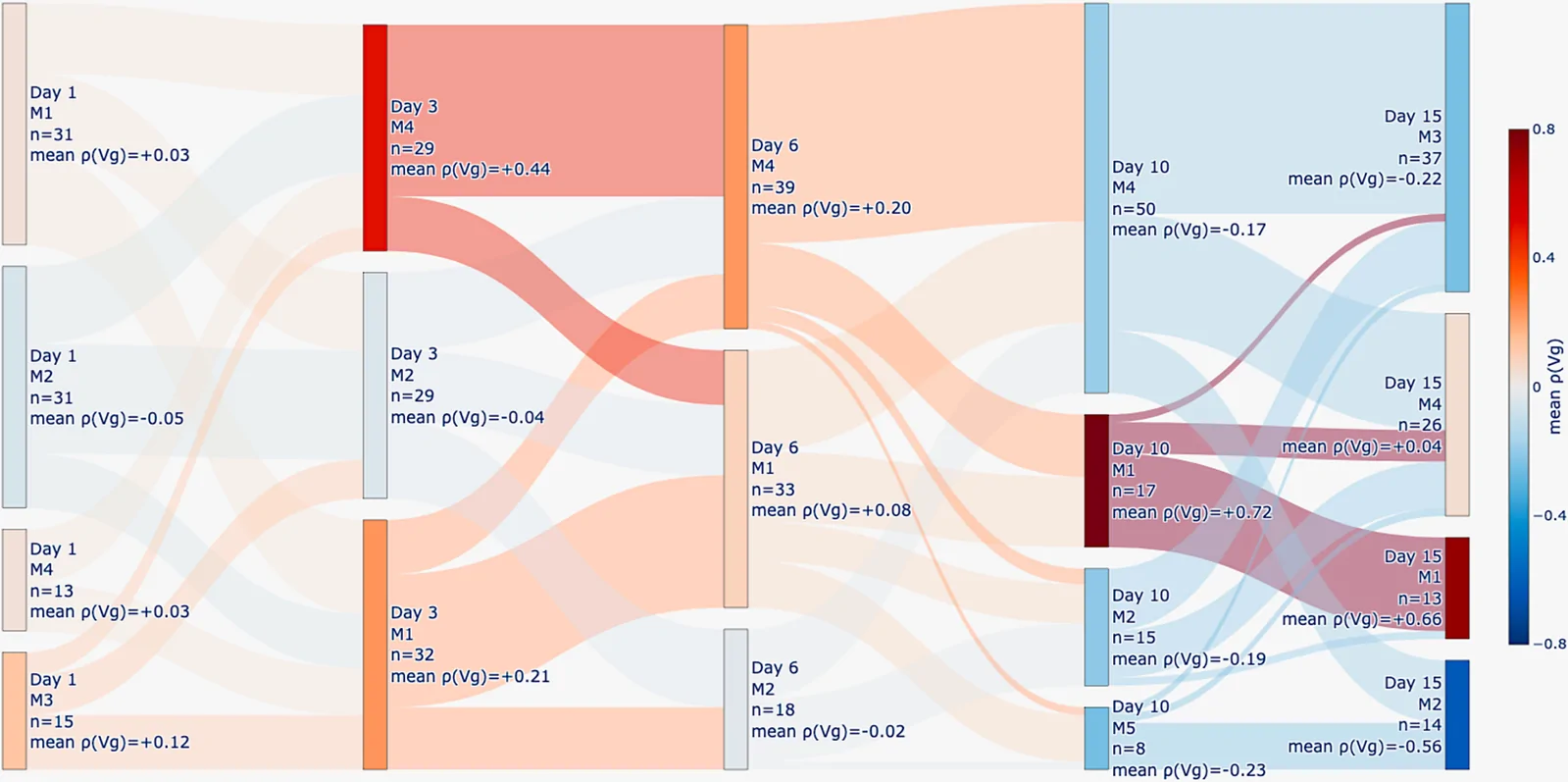
Sankey diagram showing gene membership across modules on Days 1, 3, 6, 10, and 15. Each vertical bar represents a module and is colored by its mean correlation with VG titer. Flows indicate movement of genes between modules at consecutive ages and are colored by the mean gene–VG correlation of the originating module.

A Sankey diagram provides a complementary visualization of gene movement among modules across development (Fig. 4), including the emergence of the H and L modules (M1 and M2) by Day 15.

### Vitellogenin-Centered Organization.

Correlation heatmaps of the Day 15 network reveal the bipolar organization associated with VG, with predominantly positive correlations within the H and L modules and negative correlations between them (*SI Appendix*, Fig. S3 *A* and *B*). This structure is particularly apparent when the analysis is restricted to the 27 genes comprising H and L.

**Fig. 5.**
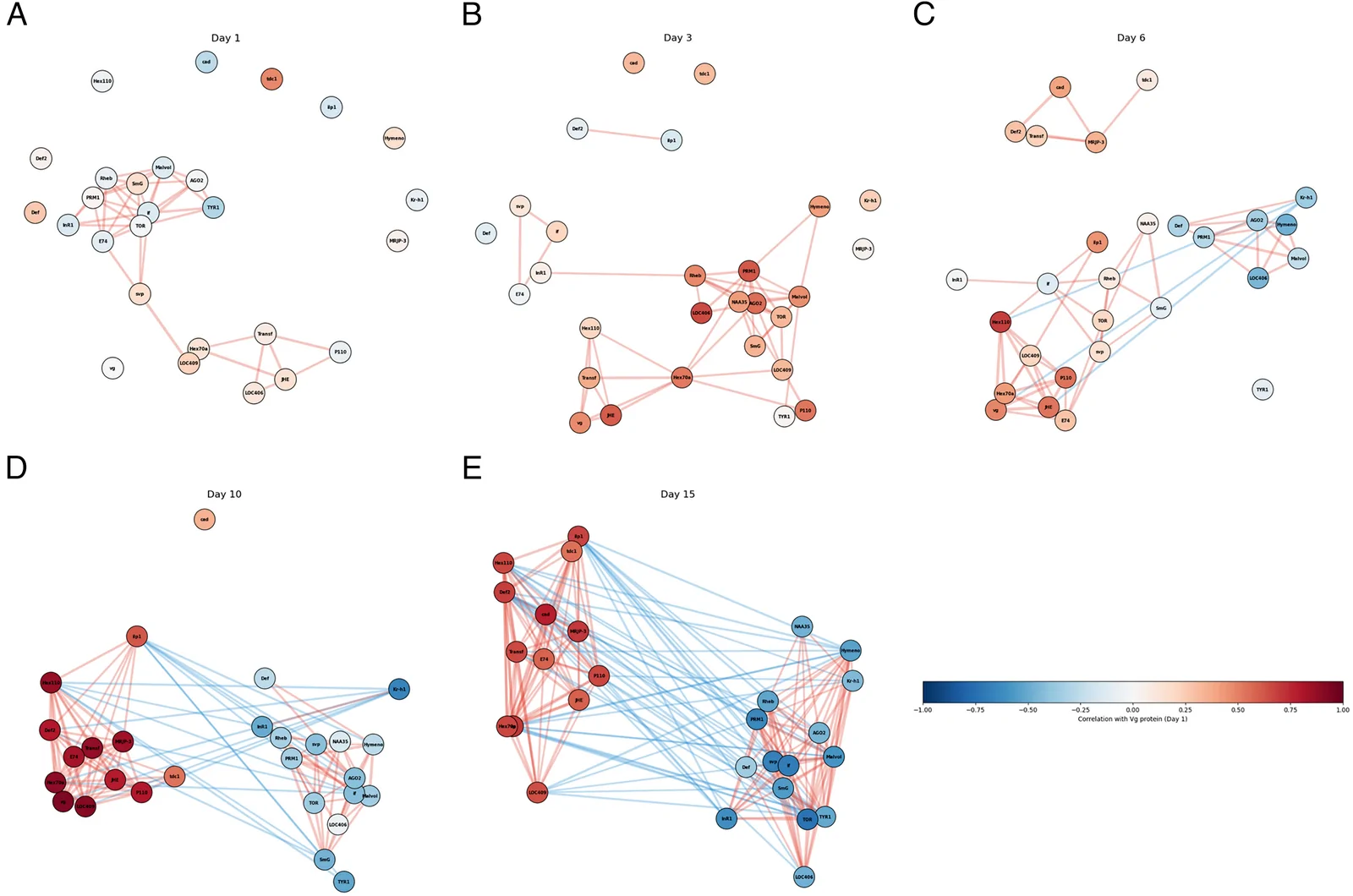
Force-directed layouts of the 27 genes comprising the H and L modules on Days 1, 3, 6, 10, and 15 (*A*–*E*; NetworkX spring_layout, k = 0.7, 50 iterations). Node colors represent correlations between gene expression and VG protein titer (red, positive; blue, negative). All displayed edges pass the BH-corrected significance threshold (q < 0.05).

Force-directed layouts illustrate the development of this organization across age (Fig. 5). Correlations among H and L genes are predominantly positive on Days 1 to 6, whereas negative correlations between the two modules emerge on Days 10 and 15 (*SI Appendix*, Fig. S4). Mean within- and between-module correlations show the same transition, beginning on Day 6 and becoming pronounced by Days 10 and 15 (*SI Appendix*, Fig. S5).

## Microstate-Level Structure

### Internal Consistency with Previous Study.

**Fig. 6.**
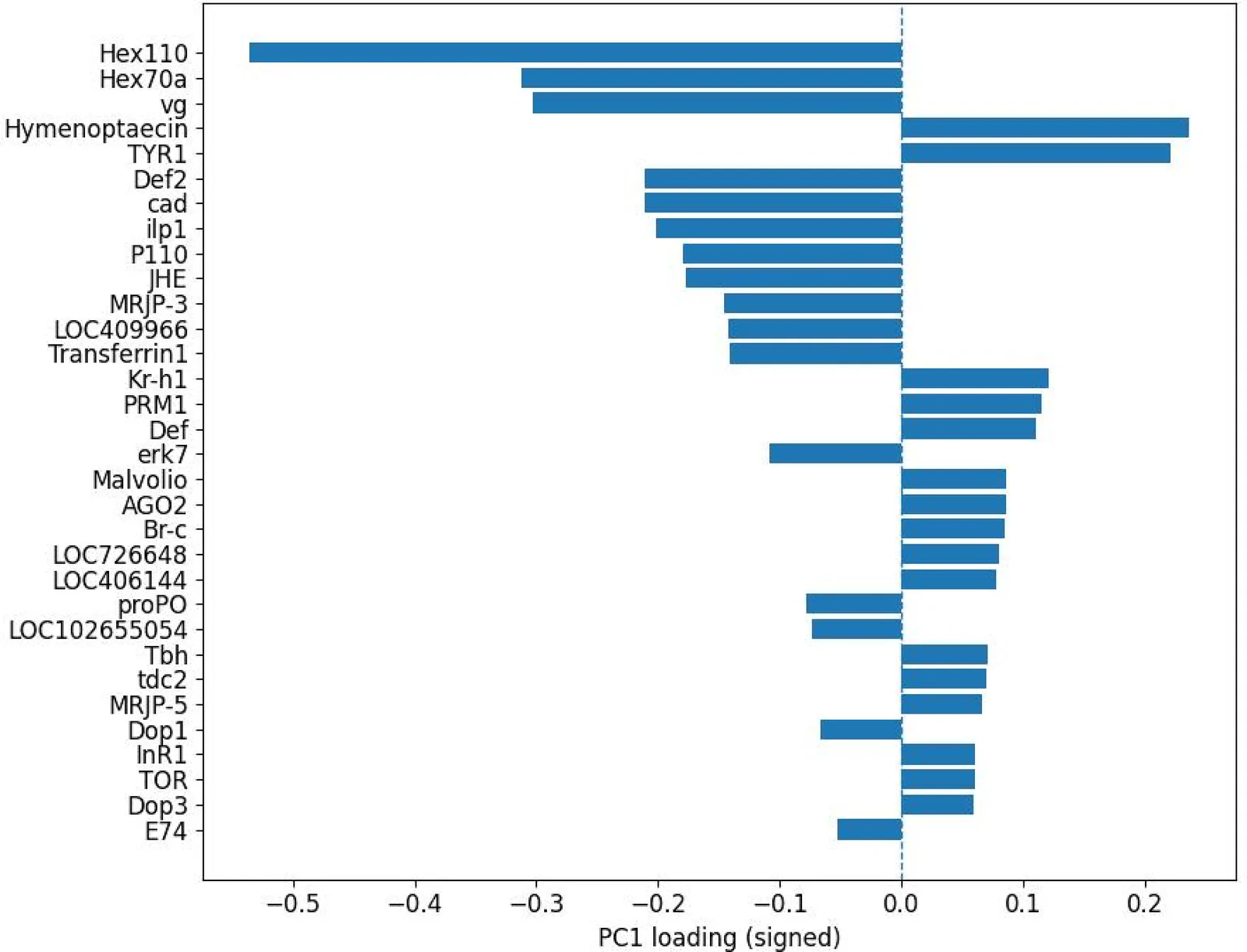
Top 32 genes ranked by absolute PC1 loading. Genes belonging to the H and L modules are concentrated among the largest loadings and have predominantly opposite signs.

A previous analysis of the same data by Daniels et al. (35) identified bistability using principal component analysis (PCA). The genes with the largest absolute PC1 loadings strongly overlap the H and L modules identified independently here (Fig. 6): 12 of 13 H-module genes have negative loadings and 10 of 14 L-module genes have positive loadings. Thus, the H and L modules largely define the principal direction of gene-expression variation, with H associated with more nurse-like and L with more forager-like expression profiles.

### Individual-Level Organization.

**Fig. 7.**
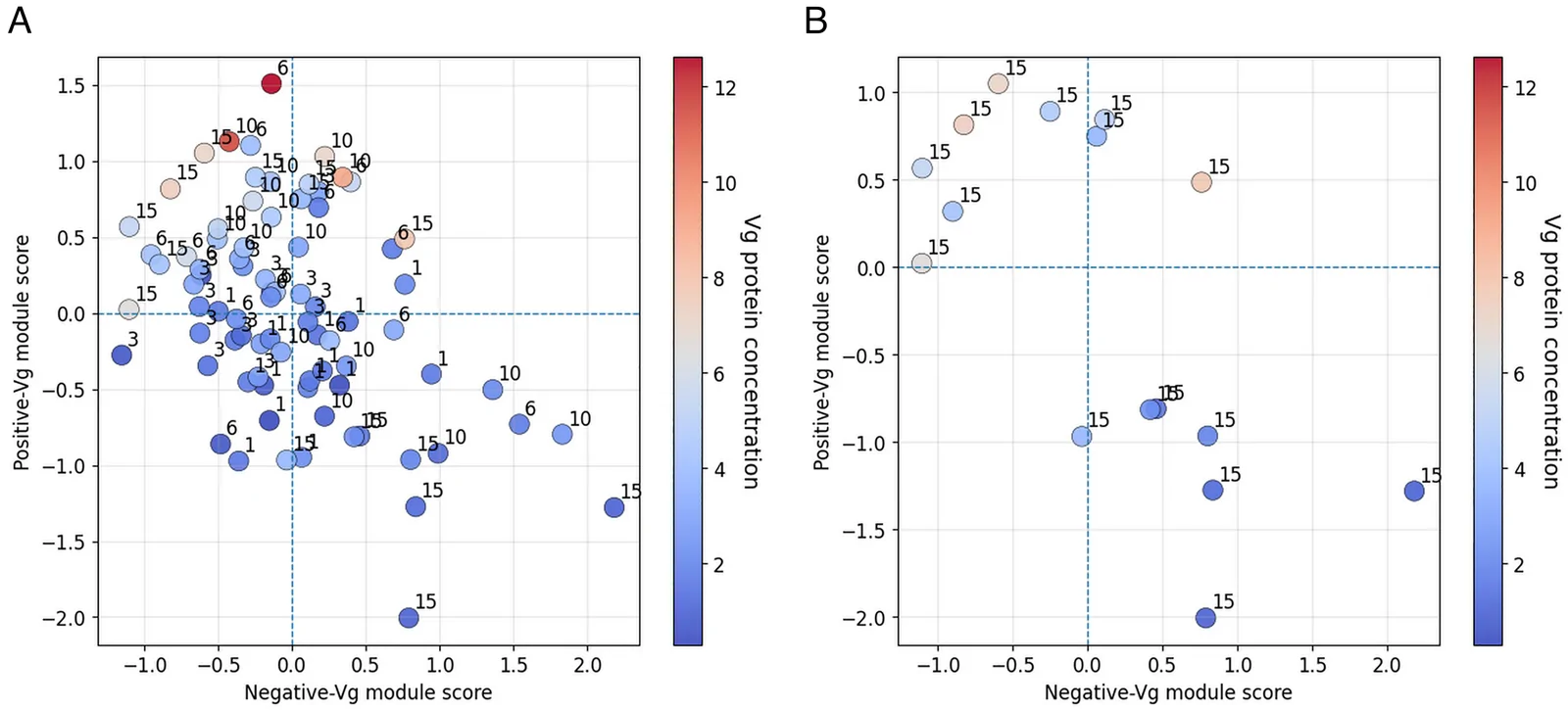
Individual bees represented by H- and L-module expression scores. Points are colored by VG protein concentration. (*A*) Bees from all ages. (*B*) Day 15 bees.

To determine how this network organization is expressed among individual bees, we calculated H- and L-module expression scores for each individual. By Day 15, bees separate into two groups characterized by relatively high expression of one module and low expression of the other (Fig. 7), consistent with two alternative VG-associated expression states.

**Fig. 8.**
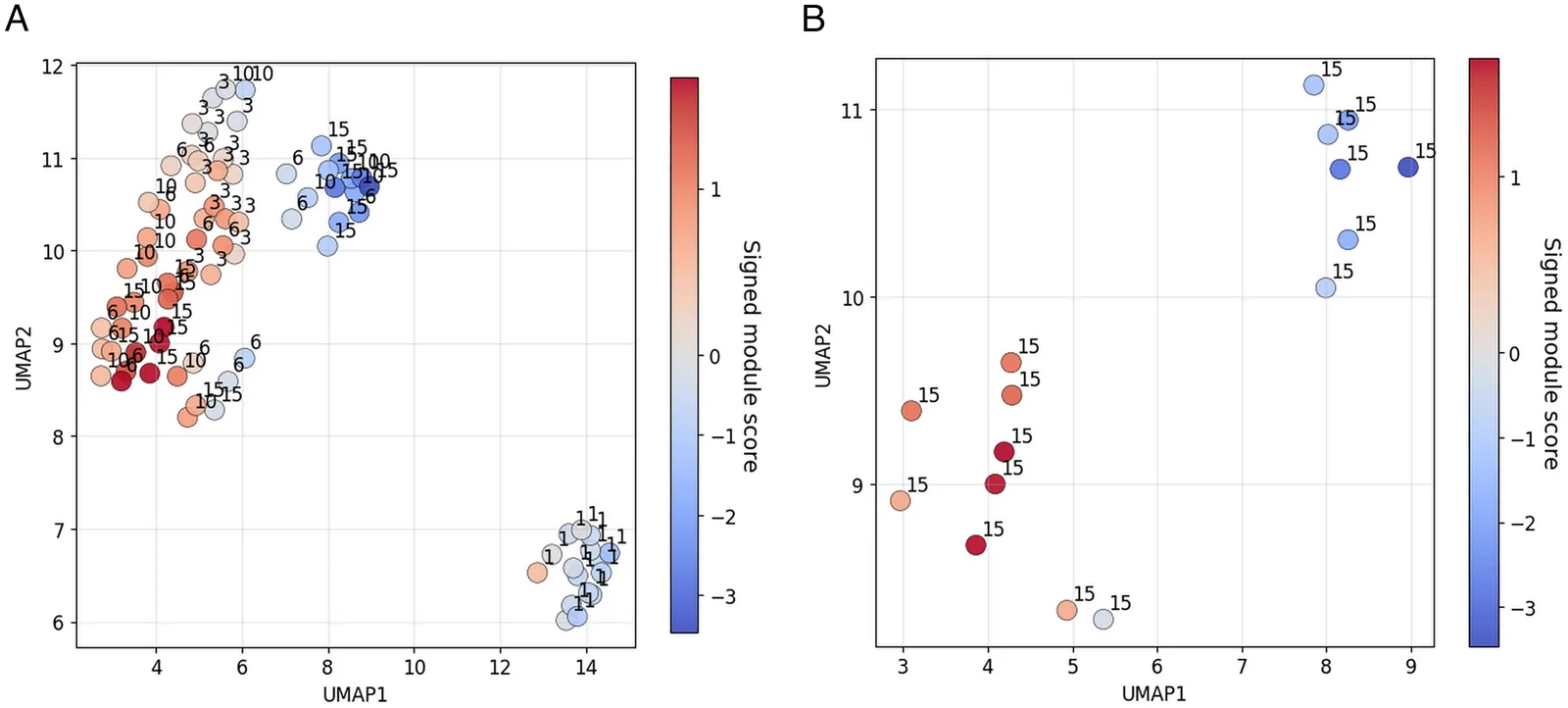
UMAP embedding of individual bees based on expression of the 27 H- and L-module genes. Points represent individual bees and are colored by signed module score. (*A*) All 80 bees across Days 1, 3, 6, 10, and 15. (*B*) Day 15 bees, showing separation into two groups with contrasting module scores.

A two-dimensional UMAP embedding of expression across the same 27 genes provides a complementary visualization (Fig. 8). Day 1 bees are distinct from older ages, whereas Day 15 bees separate into two groups corresponding to the alternative H/L expression states. Thus, although VG titer itself is not bimodal, the underlying H/L gene-expression organization differentiates into two distinct states among individual Day 15 bees.

### Microstate-Level External Validation.

We next asked whether the developmental organization identified here aligns with results of an experimental perturbation of a known regulator of worker behavioral physiology. In an independent study, Wang et al. (36) knocked down the tyramine receptor TYR1 and found coordinated changes across multiple signaling pathways, with TYR1− bees exhibiting a less forager-like molecular phenotype. Twelve genes from that study are also present in our age-series dataset, providing an independent reference against which to compare the developmental changes observed here.

**Fig. 9.**
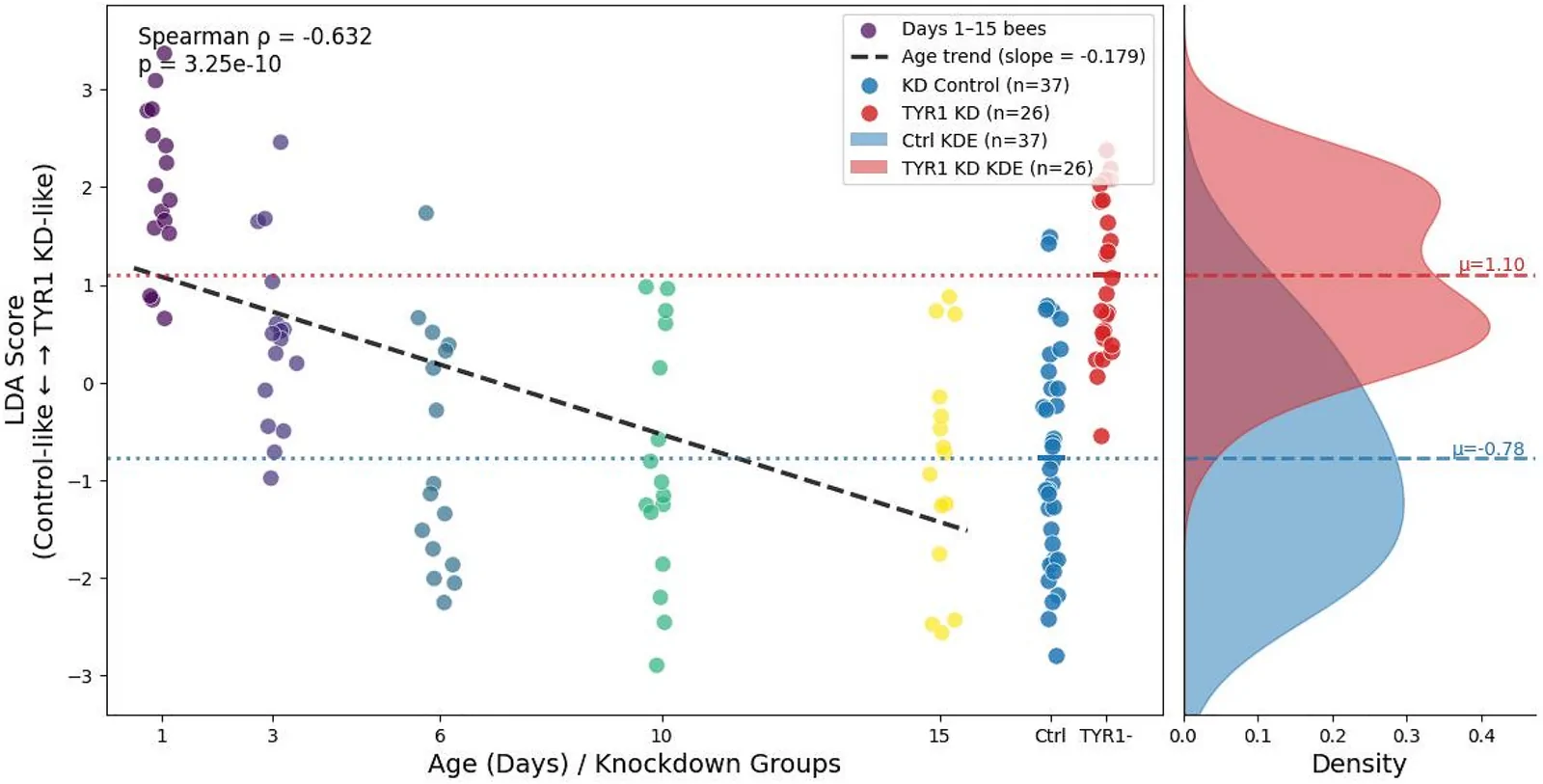
Projection of age-series bees onto the LDA axis defined by the independent TYR1 knockdown experiment. (*Left*) LDA scores for individual bees across Days 1, 3, 6, 10, and 15, together with control and TYR1− samples. Horizontal dashed lines indicate mean scores for controls (−0.776 ± 1.100, n = 37) and TYR1− bees (+1.104 ± 0.791, n = 26). LDA score decreases significantly with age (Spearman ρ = −0.632, q < 0.001). (*Right*) Distributions of LDA scores for control and TYR1− bees.

Linear discriminant analysis [LDA (14)] of these 12 genes separates TYR1− and control bees into distinct groups (Fig. 9, *Right*). Projecting bees from our age series onto this independently defined axis reveals a significant progression with age toward the control, more forager-like transcriptional state (Spearman ρ = −0.632, q < 0.001; Fig. 9, *Left*). By Day 15, mean LDA scores do not differ significantly from controls but differ significantly from TYR1− bees (*SI Appendix*, Fig. S6). Thus, the developmental transcriptional trajectory observed here aligns with an independently derived molecular axis generated by experimental perturbation of TYR1, providing external support for its association with the nurse-to-forager transition.

## Discussion

Our analyses demonstrate that the transition of worker honeybees from in-nest tasks to foraging is accompanied by a reorganization of global gene–gene correlation structure consistent with a system-level change in the dynamics of the underlying regulatory network. Rather than reflecting isolated changes in the expression of individual genes, behavioral maturation is associated with coordinated shifts in collective network properties (Figs. 1–3 and *SI Appendix*, Fig. S1), suggesting that worker behavioral states correspond to distinct macrostates of the gene regulatory system (4, 14).

This interpretation builds on earlier sociogenomic studies showing that behavioral role and social context are strongly predictive of transcriptional state in honeybees (5, 9, 14, 35–37). Subsequent work began to frame these transcriptional differences in terms of behaviorally associated gene networks (38–41) but did not examine how the global structure of gene–gene relationships reorganizes across behavioral states. By focusing on correlation structure, our results suggest that the gene network is not simply differentially regulated between nurses and foragers but reorganized as a collective system whose internal coordination changes over time.

The developmental trajectory of the correlation networks reveals a clear, multistage progression from early global coordination to later functional differentiation (Figs. 1, 3, and 4 and *SI Appendix*, Fig. S1). During early development (Days 1 to 3), gene expression patterns are highly synchronized, consistent with a unified regulatory program in which genes act in concert under shared developmental control (5, 42). At this stage, correlations are broadly distributed and largely positive (*SI Appendix*, Figs. S1 and S4), indicating exploratory coordination rather than specialization. As development proceeds (Days 3 to 6), this global synchrony weakens and distinct gene groups (modules) begin to coordinate more strongly among themselves (Figs. 2–4), marking the onset of functional differentiation (23, 41, 42).

The most pronounced reorganization occurs between Days 6 and 10, where the correlation structure undergoes a sharp transition characterized by widespread emergence of negative correlations and strong segregation between gene groups (Figs. 1–5 and *SI Appendix*, Figs. S1 and S3–S5). Previously coordinated gene programs diverge, forming internally coherent but mutually antagonistic modules (*SI Appendix*, Figs. S3–S5 and Fig. 5). This period represents a critical developmental transition in which regulatory relationships are extensively reorganized and global coordination gives way to polarized differentiation. Behaviorally, this is a period when some bees are becoming foragers while others continue to work in the nest. Comparable transitions have been reported in other biological systems, where developmental or physiological state changes correspond to high-dimensional bifurcations or critical transitions in regulatory dynamics (21, 22). In such systems, state changes are marked by coordinated restructuring of correlation patterns rather than gradual modulation of individual components (18, 19). Although our analyses identify coordinated, nonmonotonic reorganization of correlation structure consistent with state transitions, we do not directly test dynamical criteria for criticality or bifurcation [cf. Daniels et al. (35)].

Following the polarization phase, the network enters a period of relative consolidation between Days 10 and 15, during which antagonistic relationships persist but are partially softened and differentiated gene groups exhibit more stable patterns of interaction (Figs. 1–5 and *SI Appendix*, Figs. S1 and S3–S5). The system does not directly return to the globally synchronized state observed early in development; instead, within the sampled interval it exhibits a multimodule architecture that retains functional specialization while allowing renewed coordination across modules. Whether this configuration represents a longer-term stabilized state or a transitional phase enroute to subsequent reorganization cannot be determined without later time points. Similar postdifferentiation regimes have been observed in other developmental systems (20).

The late-stage bipolar organization should be interpreted in the context of behavioral heterogeneity within an aging worker cohort. The nurse-to-forager transition is not synchronous; by Days 10 to 15, some workers are transitioning toward foraging while others remain nurse-like. Consistent with this heterogeneity, individual Day 15 bees separate into alternative H- and L-associated expression states even though VG titer itself is not bimodal. The antagonistic network structure observed at the population level may therefore reflect, at least in part, differentiation among individuals occupying alternative nurse-associated and forager-associated molecular states rather than simultaneous activation of two competing programs within each bee.

The comparison with the independent TYR1 knockdown experiment provides complementary support for the biological interpretation of this developmental reorganization. The developmental trajectory observed here aligns with a transcriptional axis independently defined by experimental perturbation of TYR1, with older bees approaching the control, more forager-like state. Although this correspondence does not establish causal direction, it provides independent evidence that the network reorganization is associated with biologically meaningful variation in the nurse-to-forager regulatory system.

At the level of social organization, division of labor in insect societies has long been understood as a self-organizing process shaped by feedback between individual behavior and colony-level demands (43–45). More recent work has emphasized that collective behavior can be viewed as a form of distributed information processing, in which global system properties emerge from local interactions among individuals (46–48). Gene network organization within workers may therefore represent an additional level of collective organization associated with colony-level behavioral dynamics, rather than a sum of independent gene effects.

Notably, recent empirical work demonstrates that social interaction networks predict individual survival and task allocation in honeybees (49), highlighting the importance of relational organization at the colony level. The present results point to a parallel form of organization within the network itself, in which patterns of gene–gene coordination change systematically with behavioral role. This raises the possibility that molecular and social networks are dynamically coupled, such that transitions in colony-level organization are mirrored by coordinated reorganization of regulatory relationships within individual workers. The directions of these relationships cannot be determined from the present data. Changes in network organization may contribute to behavioral maturation, but behavioral transitions themselves may also reshape gene expression in the fat body. These processes may therefore be reciprocally coupled rather than representing a unidirectional causal pathway from network state to behavior.

Together, these findings support a shift from gene-centric explanations of behavioral maturation toward a framework that treats behavior as an emergent property of interacting regulatory systems. Our approach constrains mechanistic models of behavioral regulation by demonstrating that behavioral maturation involves coordinated reorganization of correlation structure rather than uniform changes in gene expression. Our results therefore restrict plausible mechanisms to those capable of reshaping network-level interactions among genes, a property consistent with the known system-wide actions of endocrine and neural regulators, which are associated with reshaping collective gene dynamics rather than independently controlling individual target genes (2, 6, 8)

More broadly, this work illustrates how methods developed to study collective behavior and critical transitions can be applied to network data to reveal emergent properties of gene regulatory systems underlying complex social behavior (19, 30, 50, 51). Extending such analyses to additional social contexts, developmental stages, or environmental perturbations may clarify how biological systems across different levels of biological organization exploit collective dynamics to generate flexible yet robust behavioral organization.

## Materials and Methods

### Bee Collection.

Newly emerged honeybee workers (*A. mellifera* L.) were collected, and pooled, and introduced into recipient colonies. Queens mate with a large number of haploid males, therefore, a large random sample of population genotypes were represented. Approximately 600 marked, newly emerged bees from these source colonies were introduced over a 3-d period into small nursery colonies. Frames with emerging adults from each source colony were put into incubator (37 °C). On three consecutive days 200 newly emerged bees were collected, color marked for age on the thorax and distributed evenly to the nursery colonies. To control the adult developmental environment, each nursery colony had 9 frames containing similar supplies of pollen and honey, and similar numbers and demographic distributions of brood and adult bees. Worker bees were sampled from the nursery colonies at five ages—1, 3, 6, 10, and 15 d—with 20 bees per age group collected (10 per colony). This time interval corresponds to the transition from nurse bee to forager for many, but not all, bees (1, 2).

### RNA Extraction and Gene Expression Quantification.

Complete methods of RNA extraction and gene expression quantification (see refs. 52 and 53) are described in Daniels et al. (35) (*SI Appendix*, *Materials and Methods*).

### Vitellogenin Protein Quantification.

Vitellogenin (VG) protein levels were also measured. A more detailed account is given in Daniels et al. (35). VG protein levels were measured from hemolymph samples using a published method (2). Sixteen bees per timepoint were assayed, and standardized values were used for statistical analysis.

### VG Titer Dynamics across Age.

VG titers measured in m=16 bees per age across $a\in \left\{1,3,6,10,15\right\}$ days were tested for differences in central tendency, dispersion, and trend (*SI Appendix*, *Materials and Methods*). Statistical significance of each association was assessed under the null hypothesis $H_{0}:\rho_{s}=0$ using the two-tailed *P*-value returned by scipy.stats.spearmanr (54). The edge inclusion criterion was BH-corrected q < 0.05 (see *Multiple Testing Correction* subsection below).

### Correlation Estimation and Significance Testing.

Because different individuals were sampled at each age, each day constitutes an independent cross-sectional dataset. Pairwise associations were estimated separately within each day using the Spearman rank correlation coefficient. Spearman correlation was used because, with 16 individuals per age cohort, rank-based associations are less sensitive to outliers, non-normality, and monotonic but nonlinear relationships than Pearson correlations (*SI Appendix*, *Materials and Methods*).

### Multiple Testing Correction.

To control the false discovery rate arising from the large number of pairwise statistical comparisons, all correlation *P*-values were adjusted using the BH procedure (55–59). For each day-specific network, the complete set of pairwise Spearman *P*-values (one per gene pair, totaling up to 91 × 90/2 = 4,095 tests) was ranked in ascending order and the BH q-value for the k-th ranked *P*-value was computed as q(k) = p(k) × m/k, where m is the total number of tests. An edge was retained in the network if and only if the BH-adjusted q-value satisfied q < 0.05. For gene–VG correlations, the BH correction was applied across all 90 gene tests per day. Throughout this paper, all reported significance thresholds refer to BH-adjusted q-values unless otherwise stated.

### Network Construction and Representation.

Each day-specific network was represented as an undirected graph with N=91 nodes (90 genes + VG protein). The signed adjacency matrix A (58, 60) has entries

$$A_{\mathrm{ij}}=\left\{\begin{array}{cc} w_{\mathrm{ij}}, & \text{if}\;q_{\mathrm{ij}}<0.05,\\ 0, & \text{otherwise}, \end{array}\right.$$

with $A_{\mathrm{ii}}=0$ (no self-loops) and $q_{\mathrm{ij}}$ is the q-value associated with $\rho_{s,ij}$. The absolute-weight matrix $\left|A\right|$ has entries $\left|w_{\mathrm{ij}}\right|$ for significant edges and 0 otherwise. Nodes with no significant correlations (isolates) were excluded from centrality computations.

### Signed Modularity.

Gene module assignments were obtained by applying a greedy modularity maximization algorithm to maximize the Gómez signed modularity function (*SI Appendix*, *Materials and Methods*).

### Signed Laplacian and Algebraic Connectivity.

To characterize the global organization of the signed gene correlation network, we computed the symmetric normalized signed Laplacian (59, 61–65),

$$L_{\text{sym}\;}^{\pm }=I-{\bar{D}}^{-1/2}W{\bar{D}}^{-1/2},$$

where $\bar{D}=\text{diag}\left({\bar{d}}_{1},\ldots ,{\bar{d}}_{N}\right)$ with absolute degree ${\bar{d}}_{j}=\sum_{k}\left|w_{\mathrm{jk}}\right|$. Because ${\bar{D}}^{-1/2}W{\bar{D}}^{-1/2}$ is real and symmetric, its eigenvalues are real and may be ordered

$$0\leq \lambda_{1}^{\pm }\leq \lambda_{2}^{\pm }\leq \ldots \leq \lambda_{N}^{\pm }\leq 2.$$

We used the smallest eigenvalue, $\lambda_{1}^{\pm }$, as the indicator that the network is polarized. This quantity measures the global structural integration of the signed network while accounting for both positive and negative correlations. In balanced signed networks, $\lambda_{1}^{\pm }=0$, whereas progressively larger values indicate increasing structural imbalance (frustration). Combined with the first and second spectral gaps (λ_2_± − λ1± and λ^2^± − λ^1^±) it complements signed modularity by quantifying how strongly differentiated gene modules remain integrated into a single regulatory network. Details of the spectral interpretation and the use of the spectral gap are provided in *SI Appendix*, *Materials and Methods*.

### Structural Balance Analysis.

Structural balance was assessed following Cartwright and Harary (62, 66, 67). A signed graph is balanced if its nodes can be partitioned into two sets such that all positive edges are within-set and all negative edges are between-set. At the local level, a triangle (3-clique) with edge signs $s_{12},s_{13},s_{23}$ is balanced if and only if the product of its signs is positive:

$$s_{12}\cdot s_{13}\cdot s_{23}=+1.$$

This is equivalent to requiring an even number (0 or 2) of negative edges. We performed an exhaustive census of all triangles in each day-specific network and classified them into four types: $\left(+/+/+\right),\left(+/+/-\right),\left(+/-/-\right)$, and $\left(-/-/-\right)$. The global balance ratio was computed as

$$B=\frac{T_{\text{balanced}}}{T_{\text{total}}},$$

where $T_{\text{balanced}}$ is the count of balanced triangles and $T_{\text{total}}$ is the total triangle count.

### Average Correlation across Matrices.

Mean absolute correlation was computed as the average of |r| over all BH-significant (q < 0.05) gene pairs on a given day. Mean signed correlation was computed as the average of r (with sign) over the same set of significant pairs. Trends across days were tested using the Spearman rank correlation between day index and the statistic of interest, with BH correction applied.

### Force-Directed Network Layouts (Spring Plots).

Force-directed network visualizations were generated using the Fruchterman–Reingold algorithm (68) implemented in the NetworkX spring_layout algorithm (69) (k = 0.7, 50 iterations, matplotlib visualization), which positions nodes so that strongly correlated genes appear closer together. Node positions are determined by a physical simulation in which nodes repel one another while edges act as springs that pull connected nodes together with a force proportional to the absolute value of the correlation coefficient, placing strongly correlated genes closer together. Edge thickness represents absolute correlation strength; edge color indicates sign (solid red for positive, dotted blue for negative). Node color encodes the Spearman correlation of that gene’s expression with VG protein concentration (red for positive, blue for negative). Only edges passing the BH-corrected significance threshold q < 0.05 are displayed.

### Consensus Cluster/Community Identification.

Consensus clustering is preferred over single-shot modularity optimization because at n = 16 bees per age the gene–gene correlation graph is a noisy estimate of population-level structure, and bootstrap consensus identifies the modular components that are robust to that sampling uncertainty (70–74). Details can be found in *SI Appendix*, *Materials and Methods*.

### Adjusted Mutual Information.

To quantify similarity of community partitions across days, we computed the AMI between each pair of day-specific community partitions (75). Details can be found in *SI Appendix*, *Materials and Methods*.

### Sankey Analysis of Gene Flow between Modules.

Modules identified by signed modularity optimization were aligned across consecutive days by maximizing gene overlap. The overlap matrix $O_{\mathrm{ab}}^{\left(t\to t+1\right)}=\left|M_{a}^{\left(t\right)}\cap M_{b}^{\left(t+1\right)}\right|$ was used to solve a maximum-overlap assignment problem via the Hungarian algorithm. Directed links were drawn between consecutive ages, with the weight $F_{\mathrm{ab}}^{\left(t\to t+1\right)}=\left|M_{a}^{\left(t\right)}\cap M_{b}^{\left(t+1\right)}\right|$ proportional to the number of genes flowing between modules. Each module was colored by its mean gene–VG correlation score $\phi_{a}^{\left(t\right)}=\frac{1}{\left|M_{a}^{\left(t\right)}\right|}\sum_{g\in M_{a}^{\left(t\right)}}\rho_{g,t}^{\left(VG\right)}$ using a diverging color scale centered at zero. Details can be found in *SI Appendix*, *Materials and Methods*.

### Principal Component Analysis.

PCA is a dimensionality-reduction method (76) that transforms correlated gene expression variables into a smaller set of uncorrelated components capturing the dominant patterns of variation across individuals. For each age day a, PCA was performed independently on the gene expression matrix after log transformation and gene-wise centering. The age-specific raw expression matrix $E^{\left(a\right)}\in R_{>0}^{n_{a}\times p}$ was transformed by $X_{\mathrm{ij}}^{\left(a\right)}=logE_{\mathrm{ij}}^{\left(a\right)}$, and each gene was centered across bees:

$$X_{\mathrm{ij}}^{\left(a,c\right)}=X_{\mathrm{ij}}^{\left(a\right)}-{\bar{X}}_{j}^{\left(a\right)},\;{\bar{X}}_{j}^{\left(a\right)}=\frac{1}{n_{a}}\sum_{i=1}^{n_{a}}X_{\mathrm{ij}}^{\left(a\right)}.$$

The covariance matrix was $S^{\left(a\right)}=\frac{1}{n_{a}-1}{\left(X^{\left(a,c\right)}\right)}^{⊤}X^{\left(a,c\right)}$, with eigendecomposition $S^{\left(a\right)}v_{k}^{\left(a\right)}=\lambda_{k}^{\left(a\right)}v_{k}^{\left(a\right)}$. The PC1 score for bee i at age a was $z_{i1}^{\left(a\right)}=\sum_{j=1}^{p}X_{\mathrm{ij}}^{\left(a,c\right)}v_{1j}^{\left(a\right)}$, with variance explained $\frac{\lambda_{1}^{\left(a\right)}}{\sum_{k}\lambda_{k}^{\left(a\right)}}$.

To make PC1 more interpretable, we oriented it using VG values. The Pearson correlation between PC1 scores and VG protein was computed; if positive, both scores and eigenvector were multiplied by -1, so that larger PC1 scores always correspond to lower VG protein values. Genes were ranked by $\left|v_{1j}^{\left(a\right)}\right|$; the top Y genes were retained, and the squared loading sum $M_{Y}^{\left(a\right)}=\sum_{j\in G_{Y}^{\left(a\right)}}{\left(v_{1j}^{\left(a\right)}\right)}^{2}$ measures the fraction of PC1 direction attributable to those genes. For more details see *SI Appendix*, *Materials and Methods*.

### Modules and VG Correlation Scores.

Genes from the modules obtained from consensus clustering and retained for downstream analysis are partitioned into a VG-positive set, $G_{+}$, for genes with $\rho_{g,VG}^{\left(a\right)}>0$, and a VG-negative set, $G_{-}$, for genes with $\rho_{g,VG}^{\left(a\right)}<0$. For module scoring and low-dimensional visualization, expression values for the selected genes were log-transformed before standardization. Specifically, if $x_{\text{ig}}$ denotes the observed expression value of gene g in bee i, we computed %${\bar{x}}_{\mathrm{ig}}=\log\left(1+x_{\mathrm{ig}}\right).$ Log transformation compresses the dynamic range, reduces leverage from extreme high-expression observations, and approximately stabilizes variance, making subsequent standardization and visualization more robust. After log transformation, each selected gene was standardized across all bees. Let $\mu_{g}$ and $\sigma_{g}$ denote the across-bee mean and SD of %${\bar{x}}_{\mathrm{ig}}$ for gene g. We then computed the gene-wise z-score

$$z_{\mathrm{ig}}=\frac{{\tilde{x}}_{\mathrm{ig}}-\mu_{g}}{\sigma_{g}}.$$

For each bee i, we defined a VG-positive module score and a VG-negative module score as the mean standardized expression across the two gene sets:

$$S_{i}^{\left(+\right)}=\frac{1}{\left|G_{+}\right|}\sum_{g\in G_{+}}z_{\mathrm{ig}},\;\;S_{i}^{\left(-\right)}=\frac{1}{\left|G_{-}\right|}\sum_{g\in G_{-}}z_{\mathrm{ig}}.$$

Representing bees on two-dimensional ($S_{i}^{\left(-\right)},S_{i}^{\left(+\right)}$) plot provides insight into how individual bees distribute themselves with respect to the VG gene network.

### Uniform Manifold Approximation and Projection (UMAP) (77, 78).

The two-dimensional embedding analysis was performed on the union of the Day-15 positive-VG and negative-VG consensus modules with $N_{\text{emb}\;}=27$ genes represented.

#### Expression preprocessing.

For each bee i and gene j, expression values were transformed by $\log\left(1+X_{\mathrm{ij}}\right)$ to compress dynamic range and stabilize variance. The resulting matrix was column-wise z-scored:

$$Z_{\mathrm{ij}}=\frac{\log\left(1+X_{\mathrm{ij}}\right)-\mu_{j}}{\sigma_{j}},$$

where $\mu_{j}$ and $\sigma_{j}$ are the sample mean and SD of feature j across all M=80 bees.

#### UMAP embedding.

The bee × gene matrix $Z\in R^{M\times N_{\text{emb}}}$ was projected to two dimensions by Uniform Manifold Approximation and Projection (UMAP; 77). UMAP constructs a fuzzy simplicial set representation of the k-nearest-neighbor graph in the high-dimensional feature space and optimizes a low-dimensional layout that preserves this local topology by minimizing a cross-entropy objective between the high- and low-dimensional fuzzy simplicial sets,

$$L=\sum_{i,i^{\prime }}\left[\mu_{ii^{\prime }}\log\frac{\mu_{ii^{\prime }}}{\nu_{ii^{\prime }}}+\left(1-\mu_{ii^{\prime }}\right)\log\frac{1-\mu_{ii^{\prime }}}{1-\nu_{ii^{\prime }}}\right],$$

where $\mu_{ii^{\prime }}$ is the high-dimensional fuzzy edge weight derived from local neighborhood radii and $\nu_{i^{\prime }}$ is its low-dimensional counterpart parameterized as $\nu_{i^{\prime }}={\left(1+a∥y_{i}-y_{i^{\prime }}{∥}^{2b}\right)}^{-1}$ with default smooth-curve parameters a,b. Hyperparameters were n_components =2, n_neighbors =10 (chosen as $\min\left(10,M-1\right)$, balancing local against global structure at moderate sample size), random seed 0, and otherwise default values for min_dist and metric (Euclidean). The resulting two-dimensional coordinates (UMAP $1_{i}$, UMAP $2_{i}$) were used directly for visualization, with each bee colored by its signed module score

$$s_{i}=\frac{1}{\left|M_{1}^{\left(15\right)}\right|}\sum_{j\in M_{1}^{\left(15\right)}}\;Z_{\mathrm{ij}}-\frac{1}{\left|M_{2}^{\left(15\right)}\right|}\sum_{j\in M_{2}^{\left(15\right)}}\;Z_{\mathrm{ij}}.$$

#### Implementation.

The UMAP embedding was computed with the umap-learn Python package (v0.5). Preprocessing used scikit-learn (simple Imputer for median imputation, StandardScaler for z-scoring). The pseudorandom number generator was seeded for reproducibility.

### Linear Discriminant Analysis.

LDA (78) reduces high-dimensional data using prior knowledge of group labels and identifies linear combinations of variables that best separate predefined groups by maximizing between-group variance relative to within-group variance. Two expression datasets were analyzed: i) a TYR1 knockdown dataset from Wang et al. (36) and ii) the age-series expression dataset from the present study. Gene names were harmonized across datasets by mapping assay-specific column names onto a common set of 12 overlapping genes: TYR1, vg, JHE, Kr-h1, HR46, Ftz-f1, ilp1, InR1, ILP-2, InR2, AKH, and AKHR.

Because the two datasets were generated in different experimental contexts, each gene was transformed separately within each dataset to an empirical percentile rank:

$${\tilde{x}}_{\mathrm{ig}}=\frac{r_{\mathrm{ig}}}{n},$$

where $r_{\mathrm{ig}}$ is the rank of $x_{\mathrm{ig}}$ among all samples in that dataset for gene g, and n is the number of samples. Classical Fisher LDA seeks a projection vector $w\in R^{p}$ that maximizes the ratio of between-class to within-class variance:

$$J\left(w\right)=\frac{w^{⊤}S_{B}w}{w^{⊤}S_{W}w},$$

where $S_{W}=\sum_{k=0}^{1}\sum_{i:y_{i}=k}\left(x_{i}-\mu_{k}\right)\;{\left(x_{i}-\mu_{k}\right)}^{⊤}$ is the within-class scatter matrix and $S_{B}=\left(\mu_{1}-\mu_{0}\right){\left(\mu_{1}-\mu_{0}\right)}^{⊤}$. The optimal discriminant direction satisfies $w\propto S_{W}^{-1}\left(\mu_{1}-\mu_{0}\right)$, and the scalar discriminant score for sample i is $z_{i}=w^{⊤}x_{i}$.

After fitting on the knockdown data, both the knockdown samples and the age-series samples were projected onto the same learned one-dimensional discriminant axis, so that bees from the 90-gene expression study could be scored on the control vs. TYR1 knockdown continuum and the Spearman rank correlation between LDA score and age (days posteclosion) was computed. BH correction was applied to the LDA association *P*-value. For more details see *SI Appendix*, *Materials and Methods*.

## Supplementary Material

Appendix 01 (PDF)

## Data Availability

Gene expression data and Python code used for analyses are archived at: (https://github.com/REPageJr/Expression-Data-Python-Code/) (79). Previously published data were used for this work (35).
