# Supplementary material for: System-level reorganization of gene networks underlies the nurse-to-forager transition in honeybees
Source: Proc Natl Acad Sci U S A. 2026 Oct 22;123(43):e2623939123. doi: 10.1073/pnas.2623939123 (PMC13715760; doi:10.1073/pnas.2623939123)
Supplement: Supplementary file 1 [file pnas.2623939123.sapp.pdf]

Supporting Information Appendix

System-level reorganization of transcriptomic networks underlies the nurse-to-forager transition in honey bees

Robert E. Page, Jr. and Eric Bonabeau

This file includes:

Materials and Methods

Figs. S1, S2, S3, S4, S5, S6, S7

Table S1

Supporting Information

Materials and Methods

Some of the descriptions in the article are repeated here to provide context for the statistical analyses.

RNA Extraction and Gene Expression Quantification. Complete methods of RNA extraction and gene expression quantification (1, 2)) are described in Daniels et al. (3). Total RNA was extracted from 16 randomly selected bees per age group. Samples were processed using the NanoString nCounter platform, and expression data were obtained for 90 genes. We investigated a small subset of the transcriptome because this is not a gene search but instead a search for an understanding of the function of a gene network. We determined we were more likely to understand it if we used a set of known or suspected member genes. Genes were chosen based on their known or suspected involvement in vitellogenin and juvenile hormone regulation associated with the onset of foraging behavior, nurse bee physiology related to brood food production, regulation of reproductive anatomy and physiology associated with behavioral transitions, and their localization within previously mapped quantitative trait loci associated with pollen foraging and storage behavior (Table S1).

VG titer dynamics across age. VG titers measured in $m = 16$ bees per age across $a \in \{1,3,6,10,15\}$ days were tested for differences in central tendency, dispersion, and trend. Differences in distribution across age were assessed by the Kruskal-Wallis test, with pairwise post-hoc comparisons via Dunn's test using Benjamini-Hochberg false-discovery-rate correction. Heteroscedasticity was tested by Levene's test using the median as the centring statistic (Brown-Forsythe variant). Per-age dispersion was summarised by the standard deviation, the coefficient of variation CV $= \frac{\sigma}{\mu}$, and the robust quartile coefficient of dispersion QCD $= \frac{(Q_3 - Q_1)}{Q_2}$, with the median Q_2 in the denominator. Monotone trends with age were quantified by Spearman rank correlation between age and Vg, and confirmed by the Jonckheere-Terpstra test for ordered alternatives, with normal approximation to the JT distribution. Bimodality at Days 10 and 15 was assessed by fitting one- and two-component Gaussian mixture models to $\log(1 + \mathrm{Vg})$ and comparing Bayesian Information Criterion values; $\Delta\ \mathrm{BIC} = \mathrm{BIC}_{k=1} - \mathrm{BIC}_{k=2} > 10$ was taken as strong evidence for bimodality, > 6 moderate, and > 2 weak (4). All tests were performed in Python 3.11 with 'scipy.stats' (Kruskal-Wallis, Levene, Spearman, Mann-Whitney U components of JT), 'scikit-posthocs' (Dunn's test), and 'scikit-learn' ('GaussianMixture').

Correlation Estimation and Significance Testing. Because different individuals were sampled at each age, each day constitutes an independent cross-sectional data set. Pairwise associations were estimated separately within each day using the Spearman rank correlation coefficient. Spearman correlation was used because, with 16 individuals per age cohort, rank-based associations are less sensitive to outliers, non-normality, and monotonic but nonlinear relationships than Pearson correlations. For variables X and Y measured on $m = 16$ individuals, the observations were first converted to ranks $R(X_i)$ and $R(Y_i)$, with average ranks assigned in the presence of ties. The Spearman correlation coefficient was then computed as the Pearson correlation of the rank variables,

$$\rho_s(X, Y) = \frac{\sum_i \left(R(X_i) - \overline{R(X)}\right)\left(R(Y_i) - \overline{R(Y)}\right)}{\left[\sum_i \left(R(X_i) - \overline{R(X)}\right)^2 \sum_i \left(R(Y_i) - \overline{R(Y)}\right)^2\right]^{1/2}},$$

where the sums run from $i = 1$ to m. In the absence of ties, this is equivalently

$$\rho_s = 1 - \frac{6 \sum_i d_i^2}{m(m^2 - 1)},$$

where $d_i = R(X_i) - R(Y_i)$.

Statistical significance of each association was assessed under the null hypothesis $H_0: \rho_s = 0$ using the two-tailed p-value returned by scipy.stats.spearmanr (5). The edge inclusion criterion was BH-corrected q < 0.05 (see Multiple Testing Correction subsection below).

Signed Modularity. Modules are identified by maximizing signed modularity following the formulation of Gómez et al. (6). Let $w_j^+ = \sum_k \max(w_{jk}, 0)$ and $w_j^- = \sum_k \max(-w_{jk}, 0)$ denote the positive and negative node strengths and let $w^+ = \frac{1}{2}\sum_j w_j^+$ and $w^- = \frac{1}{2}\sum_j w_j^-$ denote their totals. For a partition $C: \{1, \ldots, N\} \to \{1, \ldots, K\}$, signed modularity is

$$Q^\pm(C) = \frac{1}{2w^+ + 2w^-} \sum_{j,k} \left[w_{jk} - \left(\frac{w_j^+ w_k^+}{2w^+} - \frac{w_j^- w_k^-}{2w^-}\right)\right] \delta(C_j, C_k),$$

with $\delta(C_j, C_k) = 1$ if j and k belong to the same community and 0 otherwise. Equivalently, $Q^\pm(C) = (2w^+ + 2w^-)^{-1} \sum_c \sum_{j,k \in M_c} B_{jk}$, with the modularity matrix $B_{jk} = w_{jk} - w_j^+ w_k^+/(2w^+) + w_j^- w_k^-/(2w^-)$ and module sets $M_c = \{j: C_j = c\}$.

Greedy modularity maximization. The community partition $\hat{C}^{(a)}$ at each age is obtained by greedy agglomerative modularity maximization (7). Starting from the singleton partition with each node in its own community, at each step the algorithm merges the pair of communities (a, b) that yields the largest increase

$$\Delta Q_{ab}^{\pm} = \frac{2S_{ab}}{2w^{+} + 2w^{-}}, S_{ab} = \sum_{j \in a} \sum_{k \in b} B_{jk},$$

and terminates when no merge increases $Q^{\pm}$. The community-aggregated S matrix is updated by row-and-column addition after each merge, giving $\mathcal{O}(N^3)$ total cost without revisiting B.

Permutation null. Because greedy modularity maximization can return $Q^{\pm} > 0$ even on networks without true community structure (8), we constructed a permutation null at each age by destroying gene-gene correlations while preserving the marginal expression distributions of every gene. For each null replicate $r = 1, \dots, R (R = 200)$, each gene's expression vector $\left(X_{1,j}^{(a)}, \dots, X_{m,j}^{(a)}\right)$ was permuted independently across the 16 bees:

$$X_{i,j}^{(a,r)} = X_{\pi_j^{(r)}(i),j}^{(a)}, \pi_j^{(r)} \overset{\text{iid}}{\sim} \text{Uniform}(\mathcal{S}_m),$$

with $\mathcal{S}_m$ the symmetric group on m elements. The Spearman correlation matrix $W^{(a,r)}$ and modularity-optimal partition $\hat{C}^{(a,r)}$ were recomputed on each shuffled dataset, yielding a null distribution $\left\{Q_{\text{null}}^{\pm,r}\right\}_{r=1}^{R}$ of modularity values achievable on networks of identical size and per-gene marginals but no preserved gene-gene structure. The observed modularity was reported as a z-score relative to this null,

$$z^{(a)} = \frac{Q_{\text{obs}}^{\pm,(a)} - \mu_{\text{null}}^{(a)}}{\sigma_{\text{null}}^{(a)}},$$

with $\mu_{\text{null}}^{(a)}$ and $\sigma_{\text{null}}^{(a)}$ the mean and standard deviation of the null distribution, and as a onesided empirical p-value

$$p_{\text{emp}}^{(a)} = \frac{1}{R} \sum_{r=1}^{R} 1\left[Q_{\text{null}}^{\pm,r} \geq Q_{\text{obs}}^{\pm,(a)}\right].$$

Bootstrap confidence intervals. Sampling variability of the observed modularity estimate was quantified by a nonparametric bee-resampling bootstrap. For each bootstrap replicate

$b = 1, \ldots, B (B = 200)$, bee indices $\mathcal{J}^{(b)} = \left(i_1^{(b)}, \ldots, i_m^{(b)}\right)$ were drawn with replacement from $\{1, \ldots, m\}$, and the Spearman correlation matrix $W^{(a,b)}$ and modularity-optimal partition $\hat{C}^{(a,b)}$ were recomputed on the resampled data, yielding $Q_{\text{boot}}^{\pm,b}$. 95% confidence intervals were obtained as the empirical 2.5th and 97.5th quantiles of $\left\{Q_{\text{boot}}^{\pm,b}\right\}_{b=1}^{B}$.

Implementation. All analyses were performed in Python 3.11 using NumPy v1.24, SciPy v1.11 (scipy.stats.spearmanr, scipy.stats.rankdata), and pandas v2.0. Spearman correlations were computed by ranking each feature once per dataset and applying Pearson correlation to the rank vectors, exploiting their algebraic equivalence. The pseudorandom number generator was seeded for reproducibility.

Signed Laplacian and Spectral Balance. The symmetric normalized signed Laplacian (9, 10) is

$$L_{\text{sym}}^{\pm} = I - \bar{D}^{-1/2} W \bar{D}^{-1/2},$$

where $\bar{D} = \text{diag}(\bar{d}_1, \ldots, \bar{d}_N)$ with absolute degree $\bar{d}_j = \sum_k |w_{jk}|$. Because $\bar{D}^{-1/2} W \bar{D}^{-1/2}$ is real and symmetric, its eigenvalues are real and may be ordered

$$0 \le \lambda_1^{\pm} \le \lambda_2^{\pm} \le \cdots \le \lambda_N^{\pm} \le 2.$$

The smallest eigenvalue, $\lambda_1^{\pm}$, is used as the spectral measure of signed balance. For a connected structurally balanced signed graph, the smallest eigenvalue is zero (11). Larger values indicate increasing departure from a globally consistent two-sign organization and tension and conflicting interactions between nodes. $\lambda_1^{\pm}$ provides a system-level measure of integration, complementing modularity by capturing how well differentiated gene modules remain linked into a single regulatory system. Networks admitting a bipartition into two sets connected internally by positive and externally by negative edges yield $\lambda_1^{\pm} = 0$ exactly (11). Larger values of $\lambda_1^{\pm}$ indicate progressively more *frustrated* signed structure, in which not all correlations are mutually consistent with any binary partition. We also report the spectral gaps

$$g^{(1)} = \lambda_2^{\pm,(a)} - \lambda_1^{\pm,(a)} \text{ and}$$
$$g^{(2)} = \lambda_3^{\pm,(a)} - \lambda_2^{\pm,(a)},$$

which quantify the separation of the bipolar mode ($g^{(1)}$) from the next spectral component and the presence of additional nodes or modules that do not belong in the bipolar partition ($g^{(2)}$). When $g^{(1)}$ is close to zero, the bipolar split is strong and stable. When $g^{(2)}$ is well above zero, other nodes are not accounted for in the bipolar split.

Permutation null. Because $\lambda_{\mathrm{i}}^{\pm}$ inherit the finite-sample biases of estimated correlation matrices in the high dimensional regime $N/m > 1$ and because greedy spectral structure can arise on networks of pure noise, an interpretation of the observed eigenvalues requires comparison to a null in which gene-gene correlations are destroyed but per-gene marginal distributions are preserved. We used the same shuffling scheme as for the modularity analysis. For each null replicate $r = 1, \ldots, R (R = 200)$, each gene's expression vector at age a was permuted independently across the 16 bees,

$$X_{i,j}^{(a,r)} = X_{\pi_j^{(r)}(i),j}^{(a)}, \pi_j^{(r)\mathrm{iid}} \cup \mathrm{Uniform}(\mathcal{S}_m),$$

with $\mathcal{S}_m$ the symmetric group on m elements, yielding $W^{(a,r)}$ and $L_{\mathrm{sym}}^{\pm(a,r)}$. The eigenvalues $\lambda_{\mathrm{i}}^{\pm,(a,r)}$ and $g^{(a,r)} = \lambda_{\mathrm{i+1}}^{\pm,(a,r)} - \lambda_{\mathrm{i}}^{\pm,(a,r)}$ were recorded.

Observed values were summarized as z-scores relative to the per-age null distributions,

$$z_{\lambda_{\mathrm{i}}}^{(a)} = \frac{\lambda_{\mathrm{i,obs}}^{\pm,(a)} - \mu_{i,\mathrm{null}}^{(a)}}{\sigma_{\lambda_{\mathrm{i}},\mathrm{null}}^{(a)}}, z_g^{(a)} = \frac{g_{\mathrm{obs}}^{(a)} - \mu_{g,\mathrm{null}}^{(a)}}{\sigma_{g,\mathrm{null}}^{(a)}},$$

and as one-sided empirical p-values:

$$p_{\lambda_{\mathrm{i}}}^{(a)} = \frac{1}{R}\sum_{r=1}^{R} 1\left[\lambda_{\mathrm{i}}^{\pm,(a,r)} \le \lambda_{\mathrm{i,obs}}^{\pm,(a)}\right], p_g^{(a)} = \frac{1}{R}\sum_{r=1}^{R} 1\left[g^{(a,r)} \ge g_{\mathrm{obs}}^{(a)}\right].$$

$\lambda_{\mathrm{i}}^{\pm}$ larger than null indicates more bipolar structure than chance, $g^{(1)}$ larger than null indicates a more cleanly separated bipolar mode than chance and $g^{(2)}$ larger than null indicates the existence of additional nodes or modules not accounted for in the bipolar organization.

Bootstrap confidence intervals. Sampling variability of $\lambda_{\mathrm{i,\,obs}}^{\pm,(a)}$ and $g_{\mathrm{obs}}^{(a)}$ was quantified by a non-parametric bee-resampling bootstrap. For each replicate $b = 1, \ldots, B (B = 200)$, bee indices $\mathcal{I}^{(b)} = \left(i_1^{(b)}, \ldots, i_m^{(b)}\right)$ were drawn uniformly with replacement from $\{1, \ldots, m\}$, and the eigenvalues of $L_{\mathrm{sym}}^{\pm,(a,b)}$ were recomputed on the resampled correlation matrix. 95% confidence intervals were obtained as the empirical 2.5th and 97.5th quantiles of the bootstrap distributions of $\lambda_{\mathrm{i}}^{\pm,(a,b)}$ and $g^{(a,b)}$, respectively.

Implementation. Eigen-decompositions were computed with scipy.linalg.eigh on $L_{\mathrm{sym}}^{\pm}$ symmetrized by $L \mapsto (L + L^{\top})/2$ to suppress floating-point asymmetry. All other

infrastructure (Spearman correlations via rank-then-Pearson, permutation and bootstrap loops, random number seeding) was shared with the modularity analysis described above.

Consensus Cluster and Community Identification. We use three algorithms with explicitly tunable cluster count K are applied to each adjacency:
Signed Laplacian spectral clustering (9). Let $\bar{D} = \operatorname{diag}(\bar{d}_1, \ldots, \bar{d}_N)$ with $\bar{d}_j = \sum_k |A_{jk}|$, and define the symmetric normalized signed Laplacian

$$L_{\text{sym}} = I - \bar{D}^{-1/2} A \bar{D}^{-1/2}.$$

Let $U \in \mathbb{R}^{N \times K}$ collect the eigenvectors of L_{sym} corresponding to its K smallest eigenvalues. After row normalization

$$T_{jl} = \frac{U_{jl}}{\left(\sum_{k=1}^{K} U_{jl^k}^2\right)^{1/2}},$$

K-means is applied to the rows of T, yielding partition C^{spec} with $C_j^{\text{spec}} \in \{1, \ldots, K\}$ the cluster label of node j.

Hierarchical clustering on signed correlation distance. The dissimilarity $d_{jk} = 1 - \rho_{s,jk} \in [0,2]$ places anti-correlated nodes at maximal distance. Clusters $\mathcal{A}, \mathcal{B}$ are merged by Ward's minimum-variance criterion (13),

$$\Delta(\mathcal{A}, \mathcal{B}) = \frac{|\mathcal{A}||\mathcal{B}|}{|\mathcal{A}| + |\mathcal{B}|} \|\bar{c}_{\mathcal{A}} - \bar{c}_{\mathcal{B}}\|^2,$$

with $\bar{c}_{\mathcal{A}}, \bar{c}_{\mathcal{B}}$ the cluster centroids in the dissimilarity embedding. Cutting the dendrogram at K clusters gives partition C^{hier}.

Soft signed adjacency with topological overlap (14). The signed soft adjacency suppresses weak correlations through a power transform with $\beta = 6$,

$$\tilde{a}_{jk} = \left(\frac{1 + \rho_{s,jk}}{2}\right)^{\beta}, \tilde{a}_{jj} = 0,$$

with connectivity $\kappa_j = \sum_l \tilde{a}_{jl}$. The Topological Overlap Matrix is

$$\text{TOM}_{jk} = \frac{\sum_{l \neq j,k} \tilde{a}_{jl} \tilde{a}_{lk} + \tilde{a}_{jk}}{\min(\kappa_j, \kappa_k) + 1 - \tilde{a}_{jk}}, \text{TOM}_{jj} = 1.$$

Ward-linkage hierarchical clustering on the dissimilarity $1-\mathrm{TOM}_{jk}$, cut at K, gives partition C^{tom}.

Bee-resampling consensus. Because gene-gene correlations are estimated from $m=16$ bees per age, single-shot partitions are subject to non-trivial sampling variability. To quantify this and identify partition components that are robust to it, we apply a non-parametric bee-resampling consensus (15, 16, 17). For $b=1,\ldots,B$:

1. Draw bee resample indices $\mathcal{J}^{(b)}=\left(i_1^{(b)},\ldots,i_m^{(b)}\right)$ with $i_l^{(b)} \overset{\text{iid}}{\sim}$ Uniform $\{1,\ldots,m\}$;
2. Compute pairwise Spearman correlations and the signed adjacency $A^{(b)}$ on $\mathcal{J}^{(b)}$;
3. For each method $\mu\in\mathcal{M}=\{$ spec, hier, tom $\}$, compute the partition $C^{(b,\mu)}$ at fixed K.

The co-clustering count and consensus co-membership matrix are

$$n_{jk}^{co}=\sum_{b=1}^{B}\sum_{\mu\in\mathcal{M}}1\left[C_j^{(b,\mu)}=C_k^{(b,\mu)}\right],\Pi_{jk}=\frac{n_{jk}^{co}}{B\cdot|\mathcal{M}|}.$$

By construction Π is symmetric, $\Pi_{jk}\in[0,1]$, and $\Pi_{jj}=1$. We used $B=300$ and $|\mathcal{M}|=3$, giving 900 partitions per consensus.

Final partition and module labeling. The final partition $C^{\star}$ was obtained by Ward-linkage hierarchical clustering on the consensus dissimilarity $1-\Pi_{jk}$, cut at K clusters. Module sets are written $M_c=\{j:C_j^{*}=c\}$. To anchor labels biologically, the cluster containing the VG-protein node was relabeled M_1; remaining clusters were labeled $M_2,\ldots,M_K$ in descending order of withinmodule mean $\left|\rho_{j,\mathrm{VG}}^{(a)}\right|$.

Choice of cluster count. The cluster count K at each age was selected by sweeping over $K\in\{2,\ldots,8\}$ and combining four diagnostics:
(i) The Monti consensus-CDF area criterion (40):

$$A(K)=\int_0^1 F_K(x)dx,\Delta(K)=\frac{A(K)-A(K-1)}{A(K-1)},$$

where F_K is the empirical cumulative distribution function of the off-diagonal Π_{jk} values at cluster count K. The smallest K at which $\Delta(K)$ falls below 2% indicates that further subdivision does not sharpen the consensus structure.
(ii) The minimum module stability $\min_c S_c$ (defined below); the largest K at which $\min_c S_c\geq 0.6$ is retained.

(iii) The eigengap $\lambda_{K+1} - \lambda_K$ of the signed normalized Laplacian L_{sym} on the full-data adjacency, identifying the natural number of spectral (12).
(iv) Cross-day partition agreement, quantified by the Adjusted Mutual Information $\mathrm{AMI}\left(C^{\star,(a)}, C^{\star,(a')}\right)$ between consecutive ages (see below); K that maximizes adjacent-day AMI identifies the most temporally stable modular structure.

The selected values were $K = 5$ at age 1; $K = 3$ at ages 3 and 6; $K = 4$ at ages 10 and 15 .

Stability scoring. For each node j, the per-node stability is the mean co-clustering probability with its module mates,

$$s_j = \frac{1}{\left|M_{C_j^*}\right| - 1} \sum_{\substack{k \in M_{Cj}^* \\ k \neq j}} \Pi_{jk}.$$

For each module M_c with $|M_c| \geq 2$, the module stability is the mean pairwise co-clustering probability among its members,

$$S_c = \binom{|M_c|}{2}^{-1} \sum_{\{j,k\} \subset M_c} \Pi_{jk}.$$

Modules are reported as highly stable when $S_c \geq 0.75$, suggestive when $0.60 \leq S_c < 0.75$, and not robustly distinguishable otherwise. Within each module, nodes with $s_j \geq 0.70$ constitute the module core, $0.50 \leq s_j < 0.70$ the periphery, and $s_j < 0.50$ are reported as weakly assigned.

Adjusted Mutual Information. Following the consensus-clustering procedure described above, each age $a \in \{1,3,6,10,15\}$ yields a partition $C^{\star,(a)}$ of the $N = 90$ genes (the VG protein node was excluded from this analysis to focus on gene-gene structure) into $K^{(a)}$ modules:

$$C^{\star,(a)}: \{1, \dots, N\} \longrightarrow \left\{1, \dots, K^{(a)}\right\},$$

with module sets $M_r^{(a)} = \left\{j: C_j^{\star,(a)} = r\right\}$ for $r = 1, \dots, K^{(a)}$. The selected cluster counts were $K^{(1)} = 4, K^{(3)} = 3, K^{(6)} = 3, K^{(10)} = 4$, and $K^{(15)} = 4$.

Pairwise partition similarity. For any two ages $a \neq a'$, the agreement between their consensus partitions was quantified by Adjusted Mutual Information. Treating $C^{\star,(a)}$ and $C^{\star,(a')}$ as discrete random variables on the shared node set $\{1, \dots, N\}$, the joint contingency table has cell counts

$$n_{rc}^{(a,a')} = \left| M_r^{(a)} \cap M_c^{(a')} \right|, r = 1, \dots, K^{(a)}, c = 1, \dots, K^{(a')},$$

with marginal cluster sizes $u_r^{(a)} = \left| M_r^{(a)} \right|$ and $v_c^{(a')} = \left| M_c^{(a')} \right|$.
The empirical Shannon entropy of each partition and their mutual information are

$$H\left(C^{\star,(a)}\right) = -\sum_{r=1}^{K^{(a)}} \frac{u_r^{(a)}}{N} \log \frac{u_r^{(a)}}{N},$$
$$I\left(C^{\star,(a)}, C^{\star,(a')}\right) = \sum_{r=1}^{K^{(a)}} \sum_{c=1}^{K^{(a')}} \frac{n_{rc}^{(a,a')}}{N} \log \frac{N n_{rc}^{(a,a')}}{u_r^{(a)} v_c^{(a')}}.$$

The unadjusted normalized mutual information $\mathrm{NMI}\left(C^{\star,(a)}, C^{\star,(a')}\right) = I/\sqrt{H(C^{\star,(a)}) H\left(C^{\star,(a')}\right)}$ is not chance-corrected and is biased upward when partitions have different cluster counts, which is the case here ($K^{(a)}$ ranges from 3 to 5 across ages). To eliminate this dependence we report AMI.

Chance correction. Under the permutation null in which $C^{\star,(a)}$ and $C^{\star,(a')}$ are independently random partitions with marginal cluster sizes fixed at $\left\{u_r^{(a)}\right\}$ and $\left\{v_c^{(a')}\right\}$, contingency-table cell counts follow the generalized hypergeometric distribution

$$P_H\left(n_{rc}^{(\alpha,\alpha')}\right) = \frac{u_r^{(a)}!\left(N - u_r^{(a)}\right)! \, v_c^{(\alpha')}!\left(N - v_c^{(\alpha')}\right)!}{N! \, n_{rc}^{(\alpha,\alpha')}!\left(u_r^{(a)} - n_{rc}^{(a,a')}\right)!\left(v_c^{(a')} - n_{rc}^{(a,a')}\right)!\left(N - u_r^{(a)} - v_c^{(a')} + n_{rc}^{(a,a')}\right)!}.$$

The expected mutual information under this null is given in closed form (Hubert, L., & Arabie, P. (1985). Comparing partitions. Journal of Classification, 2, 193218) by

$$\mathbb{E}\left[I\left(C^{\star,(a)}, C^{\star,(a')}\right)\right] = \sum_{r=1}^{K^{(a)}} \sum_{c=1}^{K^{(a')}} \sum_{n_{rc}=n_{rc}^{\min}}^{n_{rc}^{\max}} \frac{n_{rc}}{N} \log \frac{N n_{rc}}{u_r^{(a)} v_c^{(a')}} \cdot P_H(n_{rc}),$$

with admissible cell-count limits $n_{rc}^{\min} = \max\left(1, u_r^{(a)} + v_c^{(a')} - N\right)$ and $n_{rc}^{\max} = \min\left(u_r^{(a)}, v_c^{(a')}\right)$. The Adjusted Mutual Information is then

$$\mathrm{AMI}\left(C^{\star_1(a)}, C^{\star_1(a')}\right) = \frac{I\left(C^{\star_2(a)}, C^{\star_1(a')}\right) - \mathbb{E}\left[I\left(C^{\star_1(a)}, C^{\star_1(a')}\right)\right]}{\frac{1}{2}\left[H(C^{\star_1(a)}) + H\left(C^{\star_1(a')}\right)\right] - \mathbb{E}\left[I\left(C^{\star_1(a)}, C^{\star_1(a')}\right)\right]}.$$

Properties. By construction, $\mathbb{E}[\mathrm{AMI}] = 0$ for two independent random partitions with the marginals of $C^{\star,(a)}$ and $C^{\star,(a')}$, regardless of N or K. The metric attains $\mathrm{AMI} = 1$ when the two partitions are identical up to relabeling of clusters. Negative values are possible if $I\left(C^{\star,(a)}, C^{\star,(a')}\right) < \mathbb{E}\left[I\left(C^{\star,(a)}, C^{\star,(a')}\right)\right]$ —that is, when partitions agree less than would be expected by chance - though such values are typically small in magnitude. Unlike NMI, AMI is unbiased with respect to the cluster counts $K^{(a)}, K^{(a')}$, making it the appropriate similarity measure when comparing partitions with different numbers of clusters across ages, as is the case here.

Implementation. The pipeline was implemented in Python 3.11 using NumPy v1.24 (31), SciPy v1.11 (30) (scipy.stats.spearmanr, scipy.cluster.hierarchy.linkage/fcluster, scipy.linalg.eigh), scikit-learn v1.3 (KMeans, adjusted_mutual_info_score, normalized_mutual_info_score, adjusted_rand_score), statsmodels v0.14 (multipletests), and pandas v2.0 (32). The pseudorandom number generator was seeded for reproducibility. . The 5×5 pairwise AMI matrix across $\{1,3,6,10,15\}$ days was rendered as a heatmap; for completeness, the analogous matrices of NMI (geometric-mean normalization) and the Adjusted Rand Index (18) were also computed and reported.

Detailed description of Sankey analysis of gene flow between signed network modules across age. Modules were identified using the consensus clustering algorithm. Because module labels are arbitrary within each independently inferred day-specific partition, modules were aligned across consecutive days by maximizing gene overlap. If $M_a^{(t)}$ is module a at day t and $M_b^{(t+1)}$ is module b at day $t+1$, the overlap matrix was defined as

$$O_{ab}^{(t\to t+1)} = \left|M_a^{(t)} \cap M_b^{(t+1)}\right|.$$

Module labels at day $t+1$ were then matched to those at day t by solving a maximum-overlap assignment problem,

$$\pi^* = \arg\max_{\pi} \sum_{a} O_{a,\pi(a)}^{(t\to t+1)}$$

using the Hungarian algorithm. Modules at day $t+1$ with no overlap-based match were assigned new labels. This alignment step does not alter the within-day partitions; it only provides a stable label correspondence for visualization across ages.

The Sankey diagram was constructed to track genes, not bees, across the sequence of day-specific signed modules. Each Sankey node corresponds to a day-specific module, e.g. "Day 3 / Module 2." The size of node $M_a^{(t)}$ is the number of genes assigned to that module,

$$N_a^{(t)} = \left|M_a^{(t)}\right|$$

Directed links were drawn only between consecutive ages. The weight of the link from module a at day t to module b at day $t+1$ was defined as

$$F_{ab}^{(t\rightarrow t+1)} = \left|M_a^{(t)} \cap M_b^{(t+1)}\right|,$$

that is, the number of genes that belonged to module a at day t and module b at day $t+1$. Thus, link thickness is proportional to the number of genes flowing between modules across adjacent ages.

Coloring modules by Vg association

To relate module dynamics to Vg biology, each gene g was also assigned a day-specific Spearman correlation with Vg protein,

$$\rho_{g,t}^{(Vg)} = \mathrm{Spearman}\left(x_{g,t}, Vg_t\right),$$

computed across the 16 bees sampled at day t. For each module $M_a^{(t)}$, a module-level Vg score was then defined as the mean gene-Vg correlation within that module:

$$\phi_a^{(t)} = \frac{1}{\left|M_a^{(t)}\right|} \sum_{g \in M_a^{(t)}} \rho_{g,t}^{(Vg)}.$$

Node colors in the Sankey diagram were mapped to $\phi_a^{(t)}$ using a diverging color scale centered at zero, so that modules with $\phi_a^{(t)} > 0$ appear more Vg -aligned and modules with $\phi_a^{(t)} < 0$ appear more anti-Vg. Links were colored according to the source module's Vg score, allowing Vg-associated and anti-Vg-associated flows to be followed across age.

Software implementation

All preprocessing and correlation calculations were performed in Python. Signed network construction used Spearman correlations computed separately within each day. Sankey diagrams were rendered as interactive flow diagrams after day-to-day module alignment by maximum overlap.

Detailed description of the principal component analysis method used. For each age day, principal component analysis (PCA) was performed independently on the gene expression matrix after log transformation and gene-wise centering. PCA identifies orthogonal linear combinations of variables that successively maximize variance, and is classically defined through the eigen-decomposition of the sample covariance matrix.

Because the code applies a natural logarithm directly to the expression values, with no pseudocount added, the procedure implicitly assumes that all analyzed expression measurements are strictly positive. Log transformation is commonly used for expression data because it compresses dynamic range and often makes variance structure more amenable to linear methods.

Let a index age day, n_a the number of bees at age a, and p the number of genes. After excluding the age column and the normalized vitellogenin protein column, the age-specific raw expression matrix was

$$\mathbf{E}^{(a)} \in \mathbb{R}_{>0}^{n_a \times p}.$$

The code transforms expression by the natural logarithm,

$$X_{ij}^{(a)} = \log E_{ij}^{(a)},$$

so that

$$\mathbf{X}^{(a)} = \log \mathbf{E}^{(a)}.$$

Each gene was then centered across bees within the same age group:

$$\bar{X}_j^{(a)} = \frac{1}{n_a} \sum_{i=1}^{n_a} X_{ij}^{(a)}, X_{ij}^{(a,c)} = X_{ij}^{(a)} - \bar{X}_j^{(a)}.$$

In matrix form,

$$\mathbf{X}^{(a,c)} = \mathbf{X}^{(a)} - \mathbf{1}\left(\overline{\mathbf{X}}^{(a)}\right)^{\top},$$

where 1 is an n_a-vector of ones.
The age-specific covariance matrix was computed from the centered log-expression matrix:

$$\mathbf{S}^{(a)} = \frac{1}{n_a - 1}\left(\mathbf{X}^{(a,c)}\right)^{\top} \mathbf{X}^{(a,c)}.$$

Because $\mathbf{S}^{(a)}$ is symmetric, its eigendecomposition can be written as

$$\mathbf{S}^{(a)} \mathbf{v}_k^{(a)} = \lambda_k^{(a)} \mathbf{v}_k^{(a)}, k = 1, \dots, p,$$

with eigenvalues ordered so that

$$\lambda_1^{(a)} \geq \lambda_2^{(a)} \geq \cdots \geq \lambda_p^{(a)} \geq 0.$$

The first principal component (PC1) direction for age a was the eigenvector associated with the largest eigenvalue:

$$\mathbf{v}_1^{(a)} = \arg \max_{\mathbf{v}:\|\mathbf{v}\|=1} \mathbf{v}^\top \mathbf{S}^{(a)} \mathbf{v}.$$

The PC1 score for bee i at age a was then computed as the projection of its centered expression profile onto this direction:

$$z_{i1}^{(a)} = \mathbf{X}_i^{(a,c)} \mathbf{v}_1^{(a)} = \sum_{j=1}^{p} X_{ij}^{(a,c)} v_{1j}^{(a)}.$$

The fraction of within-age variance explained by PC1 was

$$\mathrm{VarExplained}_{\mathrm{PC1}}^{(a)} = \frac{\lambda_1^{(a)}}{\sum_{k=1}^{p} \lambda_k^{(a)}}.$$

PCA was performed on the covariance matrix rather than on standardized z-scores; therefore, genes with larger within-age variance on the log scale had greater influence on the extracted PC1 direction.

To make the sign of PC1 biologically interpretable, the code oriented PC1 using the normalized Vg protein value measured in the same bees. Specifically, for each age day the Pearson correlation between the raw PC1 scores and Vg protein was computed:

$$r^{(a)} = \mathrm{corr}\left(z_1^{(a)}, g^{(a)}\right)$$

where $\mathrm{z}_1^{(a)} = \left(z_{11}^{(a)}, \ldots, z_{n_\mathrm{a}1}^{(a)}\right)^\top$ and $\mathrm{g}^{(a)}$ is the vector of normalized Vg protein values. If $r^{(a)} > 0$, both the scores and the eigenvector were multiplied by -1 :

$$\mathbf{v}_1^{(a)} \leftarrow -\mathbf{v}_1^{(a)}, z_1^{(a)} \leftarrow -z_1^{(a)}.$$

This orientation step exploits the sign indeterminacy of eigenvectors: $\mathbf{v}$ and $-\mathbf{v}$ define the same principal axis. After this reorientation, larger PC1 scores always corresponded to lower Vg protein values within that age group.

For each age day, genes were ranked by the absolute magnitude of their PC1 coefficients,

$$\left|v_{1j}^{(a)}\right|,$$

and the code retained the genes with the largest values. The analysis retained the top 32 genes ranked by absolute PC1 loading:

$$G_{32}^{(a)} = \mathrm{Top32}_j\left(\left|v_{1j}^{(a)}\right|\right).$$

The concentration statistic is referred to here as Top32 directional mass and is defined as the summed squared PC1 coefficients of the 32 genes with the largest absolute coefficients.

$$M_{32}^{(a)} = \sum_{j\in G_{32}^{(a)}} \left(v_{1j}^{(a)}\right)^2.$$

Because the eigenvector is unit-normalized,

$$\sum_{j=1}^{p} \left(v_{1j}^{(a)}\right)^2 = 1,$$

so $M_{32}^{(a)}$ is the fraction of total squared PC1 coefficient mass captured by the top 32 genes. Horizontal bar plots displayed the signed coefficients $v_{1j}^{(a)}$ for these genes, ordered by increasing $\left|v_{1j}^{(a)}\right|$ for visualization.

Interpretation of PC1 loadings. In this code, the reported PC1_loading for gene j is the j th element of the leading eigenvector, v_{1j}. That is an important point: these are eigenvector coefficients, not gene-PC correlations and not the scaled loadings $\sqrt{\lambda_1}v_{1j}$ sometimes used in other PCA conventions.

The PC1 score for a given bee is a weighted sum of centered log-expression values,

$$z_{i1} = \sum_{j=1}^{p} X_{ij}^{(c)} v_{1j}.$$

Accordingly, the loading v_{1j} tells how strongly gene j contributes to the PC1 axis.
A positive loading means that, all else equal, above-average expression of that gene pushes a sample toward a larger PC1 score. Because the sign of PC1 was flipped whenever necessary so that larger scores correspond to lower **Vg**, a positive final loading means that relatively higher expression of that gene is associated with the low-Vg end of the age-

specific PC1 axis. Conversely, a negative final loading means that relatively higher expression of that gene is associated with the high-Vg end of the axis.

The magnitude $|v_{1j}|$ measures how influential that gene is in defining the direction of PC1. Genes with the largest $|v_{1j}|$ are therefore the genes that most strongly structure the dominant axis of covariance within that age group. Because the coefficients are unit-normalized,

$$\sum_{j=1}^{p} v_{1j}^2 = 1,$$

the squared loading v_{1j}^2 can be interpreted as the fraction of the unit-length PC1 direction attributable to gene j. This is why summing v_{1j}^2 over the top-ranked genes provides a sensible measure of how concentrated PC1 is on a relatively small subset of genes.

Two additional interpretive points follow directly from the implementation. First, because PCA was run separately for each age day, the loadings are age-specific and describe within-day covariance structure rather than a single global developmental trajectory. Second, because the analysis used the covariance matrix without variance standardization, genes with larger within-day variance on the log scale were given more leverage in defining PC1.

Detailed description of the rank-normalized linear discriminant analysis of knockdown and age-series expression data. Two expression datasets were analyzed: (i) a TYR1 knockdown dataset and (ii) an age-series expression dataset. Gene names were harmonized across datasets by mapping assay-specific column names onto a common set of 12 overlapping genes: TYR1, vg, JHE, Kr-h1, HR46, Ftz-f1, ilp1, InR1, ILP-2, InR2, AKH, and AKHR. Samples with missing Kr-h1 values in the knockdown dataset were excluded before model fitting. The knockdown class label was encoded as

$$y_i = 1\{\text{ treatment }_i = \text{ TYR- }\},$$

so that $y_i = 0$ denotes knockdown controls and $y_i = 1$ denotes TYR1 knockdown. The age-series samples were not used to fit the classifier; instead, they were projected onto the discriminant axis learned from the knockdown data.

Rank-based normalization. Because the two datasets were generated in different experimental contexts and could differ in overall scale, each gene was transformed separately within each dataset to an empirical percentile rank. For gene g and sample i, the transformed value was

$$\tilde{x}_{ig} = \frac{r_{ig}}{n},$$

where r_{ig} is the rank of x_{ig} among all samples in that dataset for gene g, and n is the number of samples in that dataset. Ranks were assigned with scipy.stats. rankdata, which by default uses average ranks for ties. Thus, $\tilde{x}_{\text{ig}} \in (0,1]$ represents the within-dataset relative position of each sample for each gene rather than its raw expression magnitude.

Let $p = 12$ denote the number of overlapping genes after harmonization. The knockdown design matrix was

$$X^{(\text{KD})} \in \mathbb{R}^{n \times \text{x} \times p},$$

and the age-series matrix was

$$X^{(\text{Age})} \in \mathbb{R}^{n_{\text{Age}} \times p},$$

with both matrices containing the rank-normalized gene values $\tilde{x}_{ig}$.
*Linear discriminant analysis***.** A one-dimensional linear discriminant analysis (LDA) model () was fit on the knockdown dataset using sklearn.discriminant_analysis.LinearDiscriminantAnalysis (n_components =1) with the default SVD solver. In scikit-learn, n_components affects the transform step and is constrained by $\min(n_{\text{classes}} - 1, p)$; because there were two classes, the maximal discriminant dimensionality was one. By default, class priors were inferred from the observed class proportions in the training data. The default SVD solver performs both classification and dimensionality reduction and does not require explicit computation of the covariance matrix.

For two classes, classical Fisher LDA seeks a projection vector $w \in \mathbb{R}^p$ that maximizes the ratio of between-class to within-class variance. Writing the class means as

$$\mu_k = \frac{1}{n_k} \sum_{i, y_i = k} x_i, k \in \{0,1\},$$

and the within-class scatter matrix as

$$S_W = \sum_{k=0}^{1} \sum_{i: y_i = k} (x_i - \mu_k)(x_i - \mu_k)^\top,$$

the Fisher criterion is

$$J(w) = \frac{w^\top S_B w}{w^\top S_W w},$$

where, for the two-class case,

$$S_B = (\mu_1 - \mu_0)(\mu_1 - \mu_0)^\top.$$

The optimal discriminant direction satisfies

$$w \propto S_W^{-1}(\mu_1 - \mu_0),$$

and the scalar discriminant score for sample i is

$$z_i = w^\top x_i.$$

The numerical solution was obtained through the SVD-based scikit-learn implementation rather than by explicitly forming S_W^{-1}.

After fitting on the knockdown data, both the knockdown samples and the age-series samples were projected onto the same learned one-dimensional discriminant axis:

$$z_i^{(\mathrm{KD})} = f_{\mathrm{LDA}}\left(x_i^{(\mathrm{KD})}\right), z_j^{(\mathrm{Age})} = f_{\mathrm{LDA}}\left(x_j^{(\mathrm{Age})}\right).$$

By construction, more negative or positive values on this axis indicate greater similarity to one or the other knockdown-trained expression state, depending on the fitted orientation of the discriminant vector.

Density visualization of discriminant scores. To compare the distributions of discriminant scores between knockdown controls and TYR1 knockdown samples, one-dimensional kernel density estimates were computed with scipy.stats.gaussian_kde. For a set of scores $\{z_i\}_{i=1}^n$, the kernel density estimate is

$$\hat{f}_h(z) = \frac{1}{nh}\sum_{i=1}^{n} K\left(\frac{z - z_i}{h}\right),$$

where K is the Gaussian kernel and h is the bandwidth. In gaussian_kde, automatic bandwidth determination is used by default, with Scott's rule

$$h \propto n^{-1/(d+4)},$$

where d is the dimensionality of the data. In this analysis, KDEs were used only for visualization of the class score distributions.

Association between discriminant score and age. To test whether the age-series samples shifted systematically along the knockdown-derived discriminant axis, the projected LDA scores were correlated with age in days using Spearman's rank correlation coefficient. For paired observations $(\boldsymbol{A_i}, \boldsymbol{z_i})$, where $\boldsymbol{A_i}$ is age and $\boldsymbol{z_i}$ is the projected LDA score, Spearman's coefficient was computed as the Pearson correlation of the rank-transformed variables:

$$\rho_s = \frac{\sum_{i=1}^{n} \left(R_{A,i} - \bar{R}_A\right)\left(R_{z,i} - \bar{R}_z\right)}{\left[\sum_{i=1}^{n} \left(R_{A,i} - \bar{R}_A\right)^2 \sum_{i=1}^{n} \left(R_{z,i} - \bar{R}_z\right)^2\right]^{1/2}},$$

where $R_{A,i}$ and $R_{z,i}$ are the ranks of A_i and z_i, respectively. The corresponding P value was obtained from scipy.stats. spearmanr. Spearman correlation is a nonparametric measure of monotone association and was used here because age takes only five ordered values with repeated samples per age group.

Graphical display. First, KDE curves were plotted for the LDA scores of knockdown controls and TYR1 knockdown samples, with dashed vertical lines marking the class means. Second, age series LDA scores were plotted against age, with a small horizontal jitter added to the age coordinate for visual separation of points:

$$A_i^* = A_i + \varepsilon_i, \varepsilon_i \sim \mathcal{N}(0, 0.15^2).$$

A least-squares linear trend line was added for visualization using numpy. polyfit, but inference on age dependence was based on the Spearman correlation described above.

Bootstrapped LDA (Fig. S6).

Datasets and gene harmonization. Two qPCR datasets were combined for this analysis: (i) a TYR1 knockdown experiment, with a control group and a TYR1-knockdown group [n = 37 and n = 26 samples respectively), and (ii) an age-series in which bees were sampled at days 1, 3, 6, 10, and 15 (n = 80 total). The intersection of genes assayed in both experiments (TYR1, *vg*, JHE, *Kr-h1*, HR46, *Ftz-f1*, *ilp1*, InR1, ILP-2, InR2, AKH, and AKHR (12 genes)) was retained.

Rank normalization. The two datasets were generated under different protocols and reported on incomparable absolute scales (relative quantification in the knockdown experiment versus raw transcript counts in the age-series). To place them on a common

dimensionless axis without imposing distributional assumptions, each gene was rank-normalized within each dataset to percentile ranks in [0, 1].

Linear discriminant analysis. A linear discriminant analysis (LDA (19); scikit-learn LinearDiscriminantAnalysis, default solver, single component) was fitted on the rank-normalized knockdown data using treatment (control vs. TYR1−) as the class label. The age-series samples were then projected onto the resulting one-dimensional discriminant axis. By construction, positive scores correspond to a knockdown-like transcriptional profile.

Stratified bootstrap. To obtain confidence intervals for per-group mean LDA scores and for pairwise contrasts between groups we performed a stratified non-parametric bootstrap with N = 2,000 iterations (seed = 0). In each iteration:

1. The knockdown samples were resampled with replacement, stratified by treatment, preserving the original control and TYR1− group sizes.
2. The age-series samples were resampled with replacement, stratified by day, preserving the original per-day group sizes.
3. Each resampled dataset was independently rank-renormalized, so that ranks reflected only within-resample structure and did not leak information from the original data.
4. A new LDA was fitted on the resampled knockdown data, and both resampled datasets were projected onto its discriminant axis.
5. Because the orientation of an LDA axis is identifiable only up to sign, the axis was sign-aligned in each iteration so that the resampled TYR1− group had a higher mean projection than the resampled control group.
6. Mean LDA scores were recorded for control, TYR1−, and each of the five day groups.

This scheme propagates uncertainty arising from sample composition, rank normalization, and LDA fitting uniformly into the confidence intervals of all seven group means, so that within-knockdown comparisons and cross-dataset comparisons are placed on the same inferential footing.

Confidence intervals and contrast tests. For each group, the 95% confidence interval was taken as the [2.5%, 97.5%] percentiles of the bootstrap distribution of group means. Point estimates were taken from the LDA fit on the full, un-resampled data, and the bootstrap distributions are reported around those estimates.

Five pairwise contrasts were specified *a priori*:

- aging effect: Day 15 − Day 1, difference in LDA scores is observed but does not reach statistical significance.

- knockdown effect: TYR1− / control, significant LDA score difference
- Day 15 vs. control, no significant difference
- Day 15 vs. TYR1−, statistically significant difference
- Day 1 vs. control, statistically significant difference

For each contrast, the bootstrap distribution of the difference in group means across the 2,000 iterations was computed. 95% percentile confidence intervals were obtained from this distribution, and two-sided bootstrap p-values were computed as $p = 2 \cdot \min[\Pr(\Delta \geq 0), \Pr(\Delta \leq 0)]$, floored at $1/N = 5 \times 10^{-4}$ to reflect the finite resolution of the resampling distribution.

We note that the within-knockdown contrast (TYR1− / control) is expected to be optimistic because the LDA direction was trained specifically to separate those two classes; refitting the model in every bootstrap replicate propagates sampling uncertainty but does not convert that contrast into an out-of-sample test. The age-series samples were not used to fit the discriminant axis and are therefore out of training sample with respect to axis estimation. However, contrasts between an age group and either training class still use a reference endpoint that contributed to fitting the axis and should be interpreted as projection-based similarity contrasts rather than fully independent validation tests.

Supplementary figures

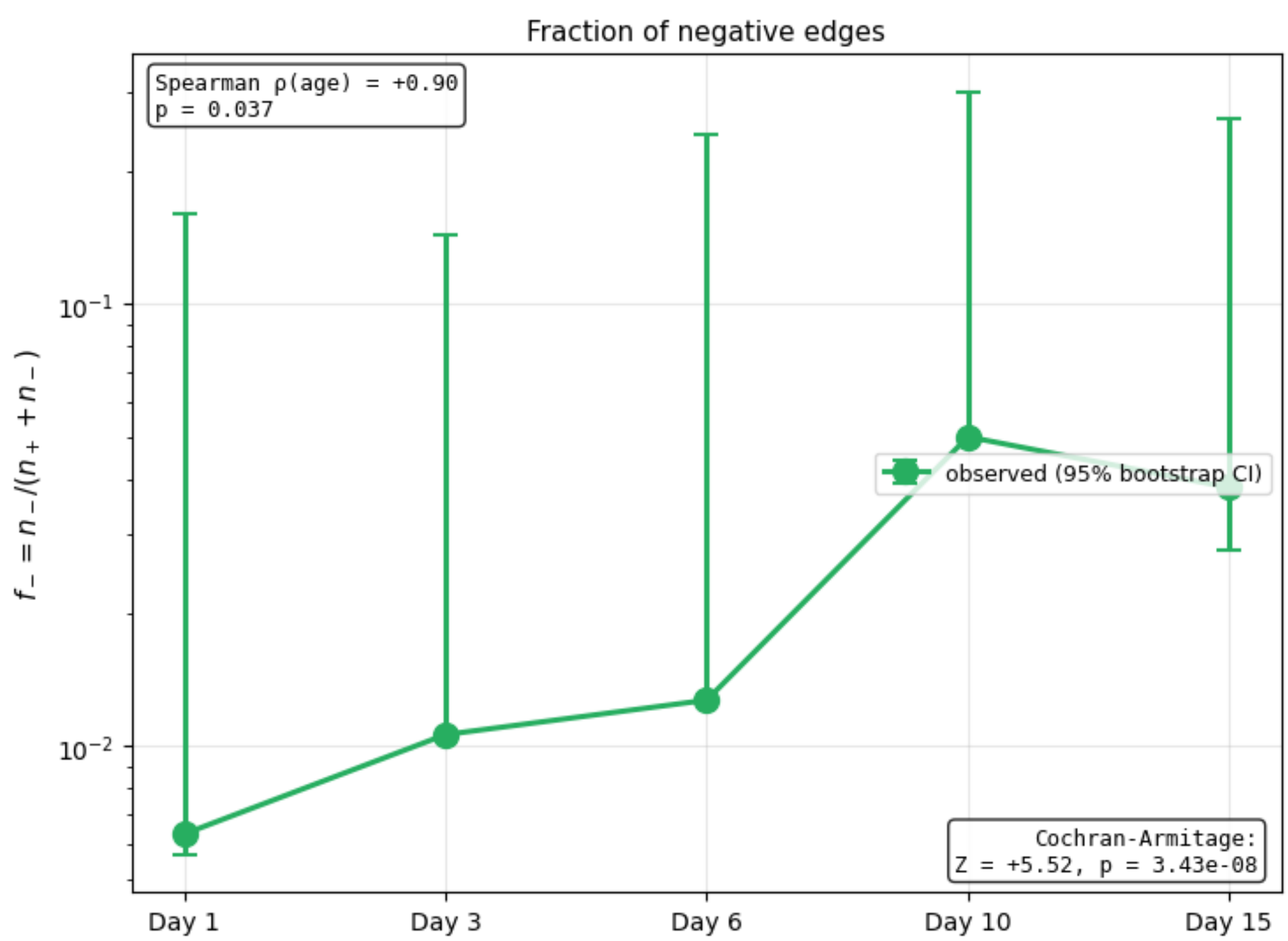

Figure S1. Fraction of negative edges (log scale). Values rise from 0.006 at Day 1 to 0.050 at Day 10 (Spearman $S = +0.90, p = 0.037$ over 5 ages; Cochran-Armitage $Z = +5.52$, $\mathrm{p} = 3.4 \times 10^{-8}$ over the underlying edge counts). The bootstrap CIs (not represented) are wide because the absolute count of negative edges is small, but the trend is highly significant when the underlying 3000 BH-significant edges are pooled across the trend test.

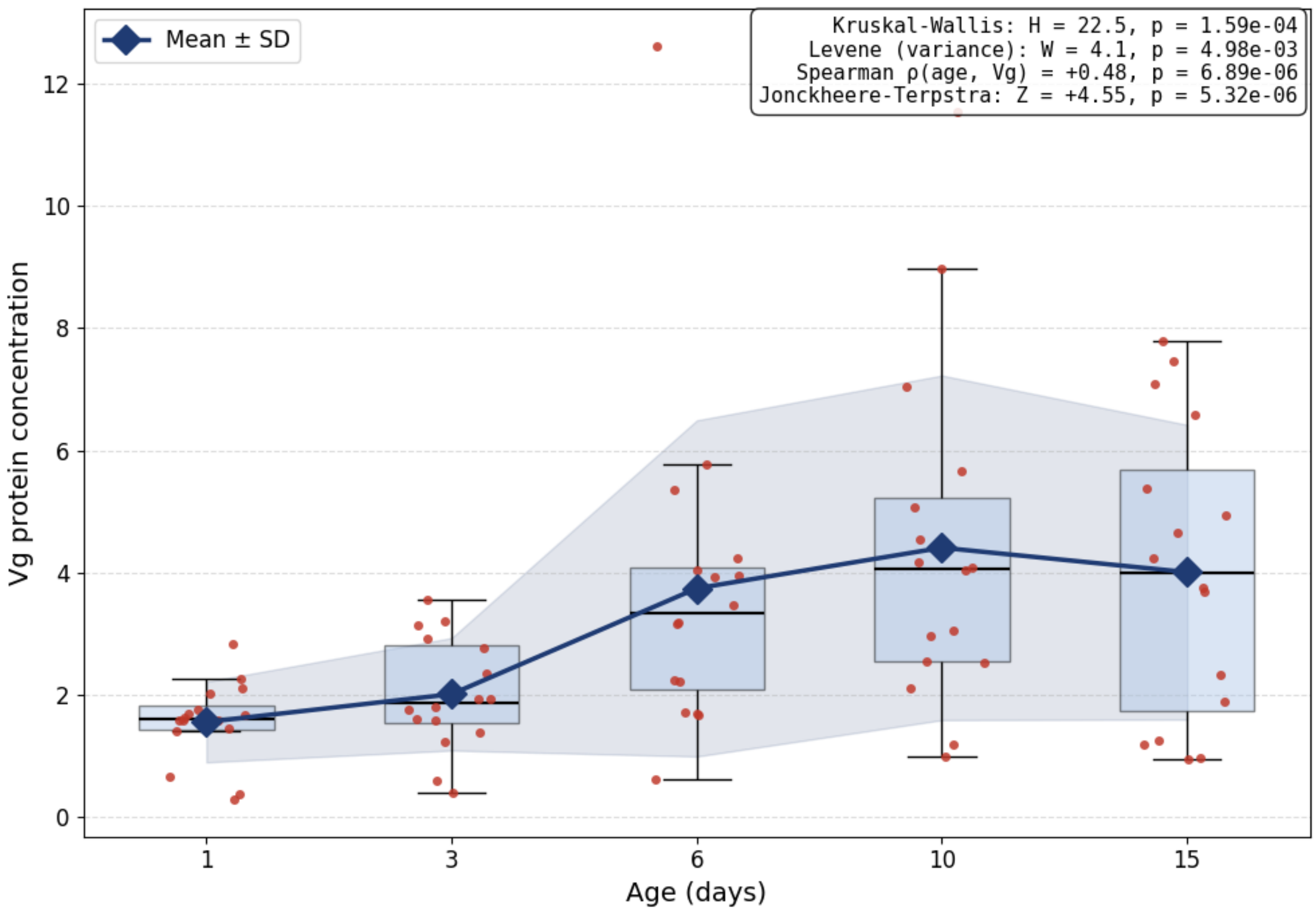

Figure S2. VG titer dynamics and dispersion across age. VG titer is measured in $n = 16$ honey-bee workers per age. Individual bees are shown as red points (jittered horizontally for visibility), with boxplots indicating the median (black horizontal line), interquartile range (box), and $1.5 \times 1\mathrm{QR}$ whiskers. Mean values are shown as dark blue diamonds connected by a line, with the shaded ribbon indicating $\pm 1\mathrm{SD}$.

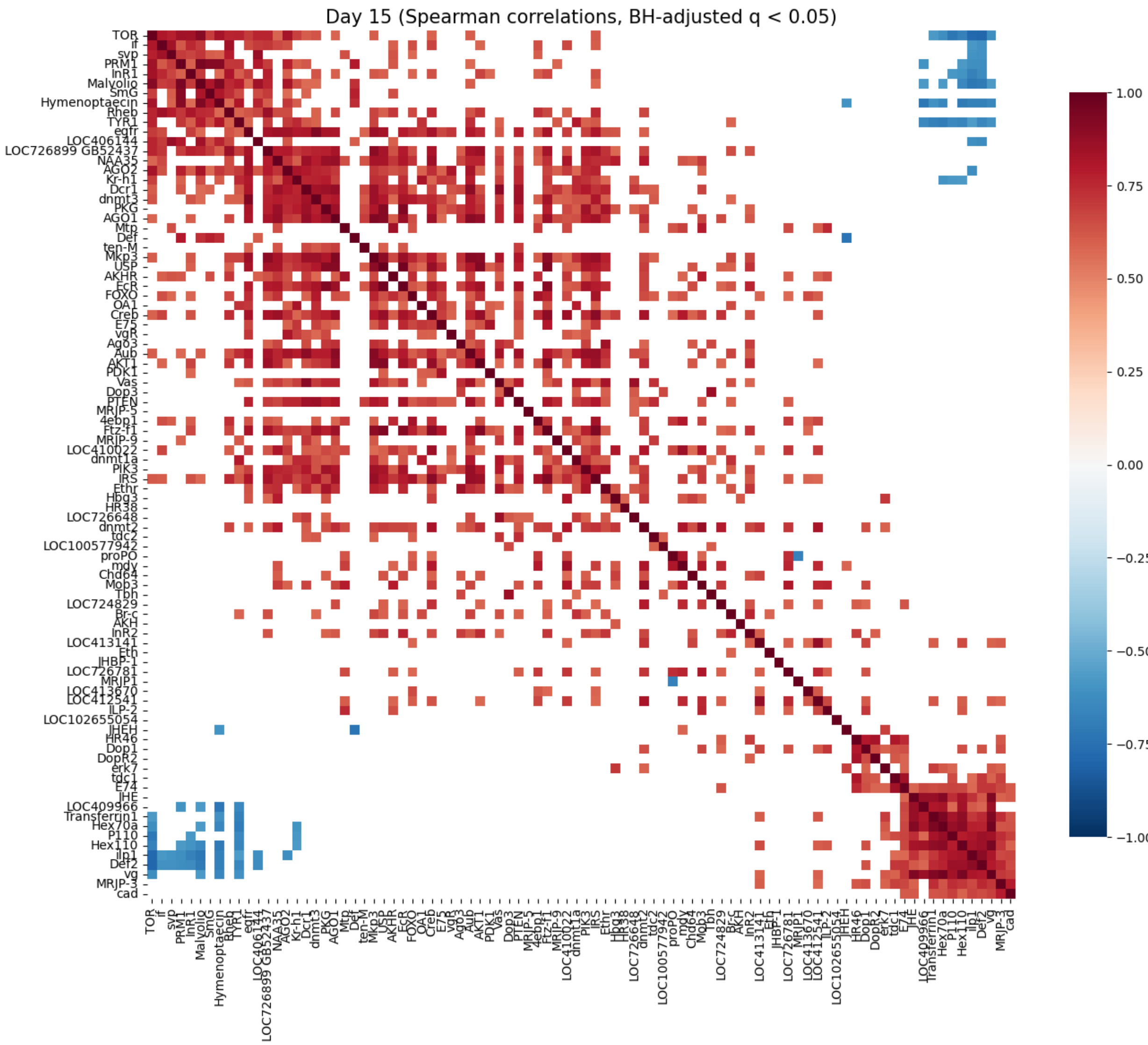
Day 15 (Spearman correlations, BH-adjusted q < 0.05)
1.00
0.75
0.50
0.25
0.00
−0.25
−0.50
−0.75
−1.00

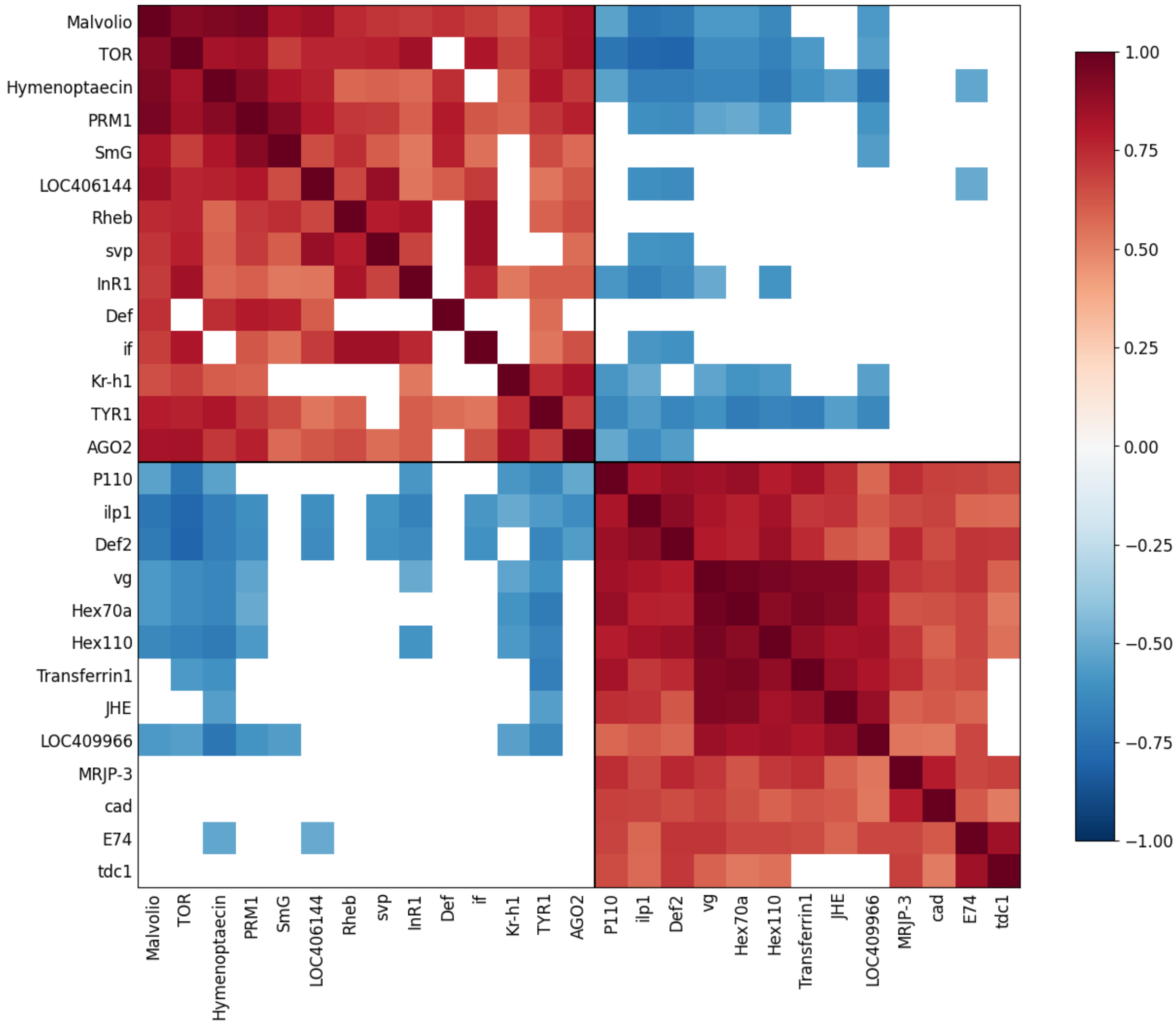

Figure S3A (upper). Correlation Matrix for all genes on Day 15 ranked by their expression levels' correlations to VG titer (in ascending order from negative to positive). All edges shown pass BH-corrected significance threshold q < 0.05. Non-significant correlations are represented as 0 (white). The nature of the modules at Day 15 can be inferred from the correlation structure: a negative VG correlation module (L) (top left corner), a positive VG correlation module (bottom right) (H) and a set of "neutral" genes with low or no significant correlation with VG. Most of the genes in either module L or H are correlated negatively (blue)to genes of the other module and positively (red) to genes of the same module.
Figure S3B (lower). Correlation Matrix on Day 15 for the 27 genes of Modules H (bottom right) and L (top left) ranked by their expression levels' correlations to VG titer (in ascending order from negative to positive). All edges shown pass BH-corrected significance threshold q < 0.05. Non-significant correlations are represented as 0 (white). Restricting analysis to

the genes from Day 15's modules H and L reveal the same antagonistic structure as shown in Fig. S3A, but with increased clarity and separation.

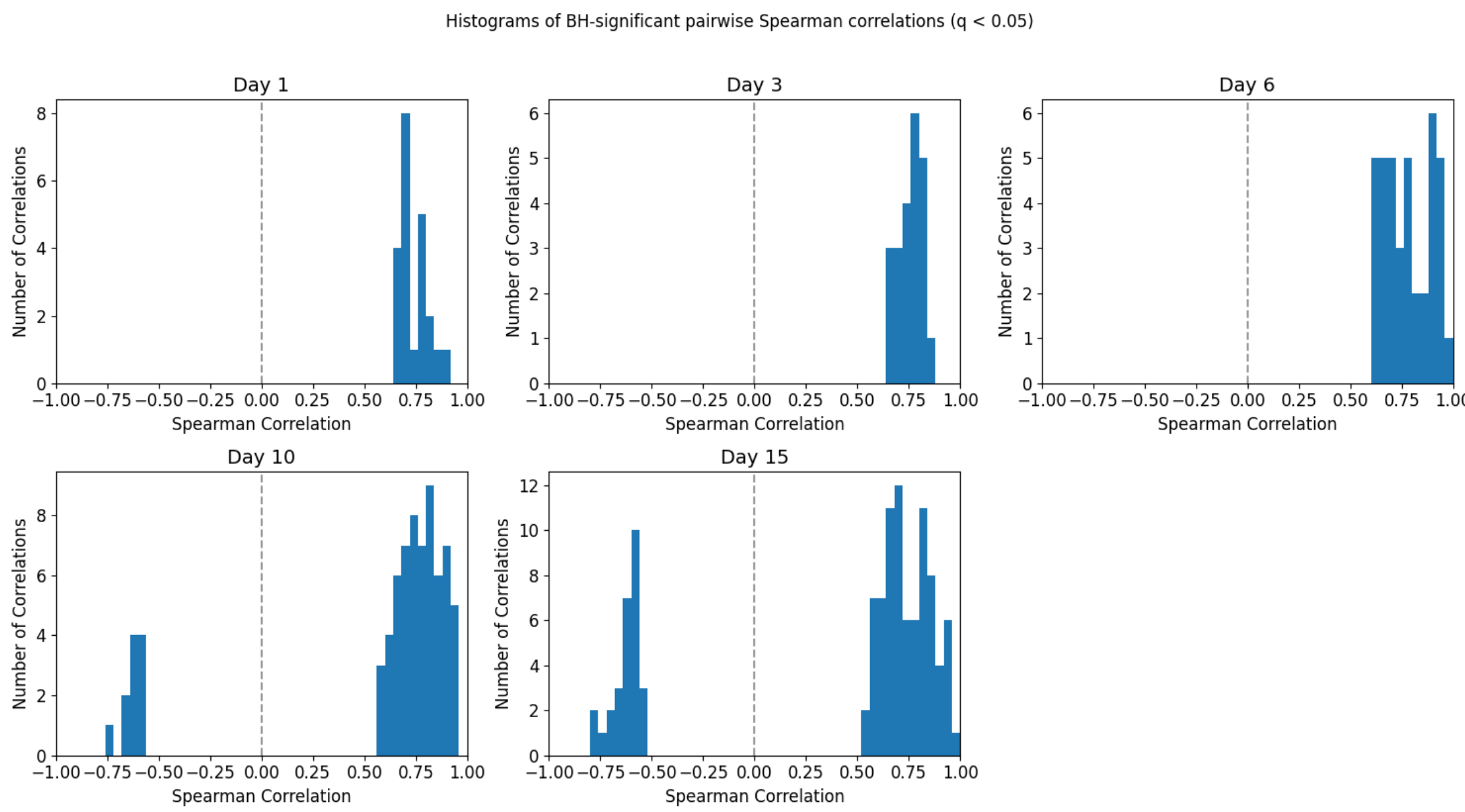

Figure S4. Histogram of significant gene-gene correlation values (BH q<0.05) on Days 1, 3, 6, 10 and 15.

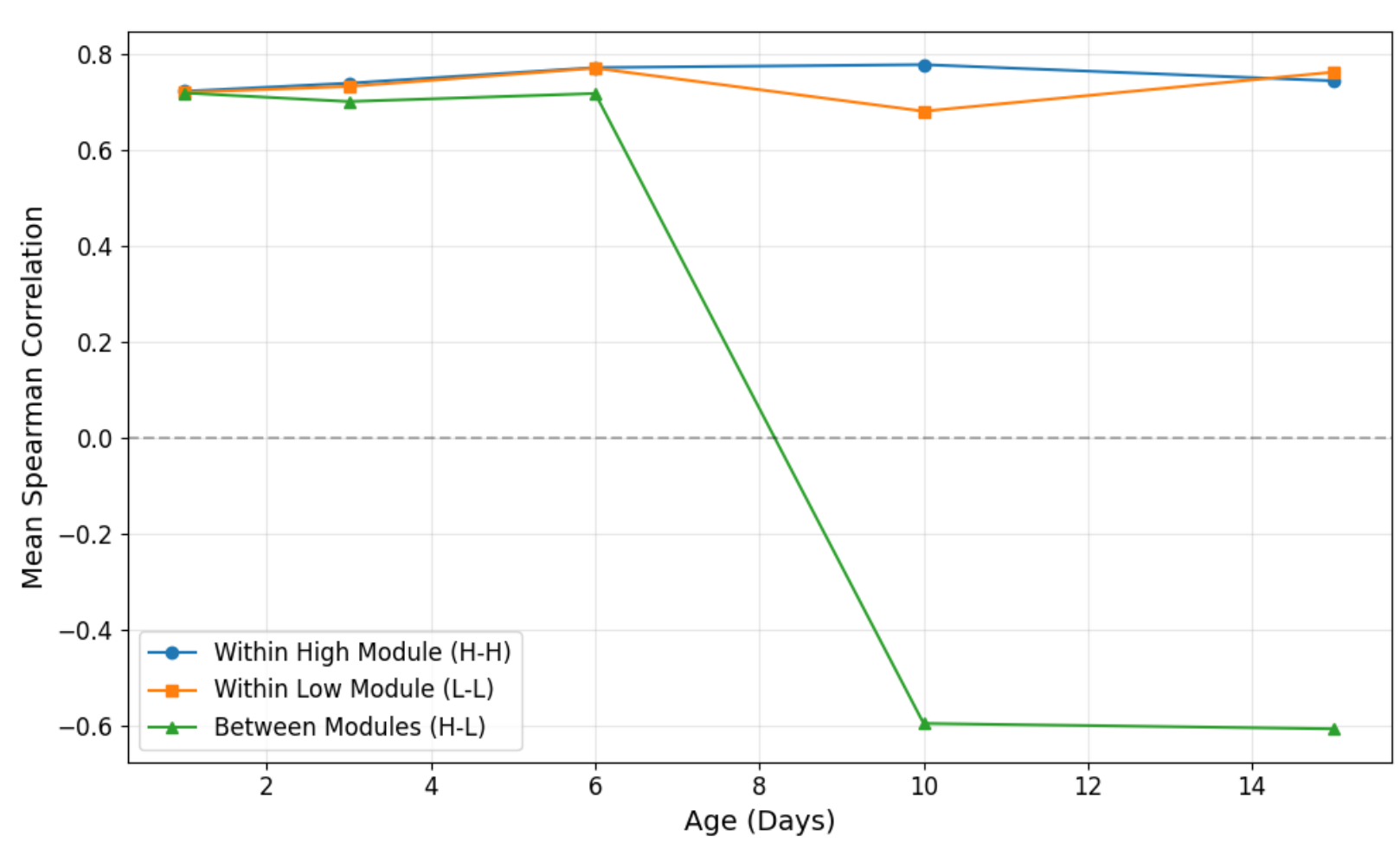

Figure S5. Mean of significant BH-corrected correlation values (q<0.05) of genes within and between modules L and H on Days 1, 3, 6, 10 and 15. An antagonistic relationship emerges between L and H between Days 6 and 10.

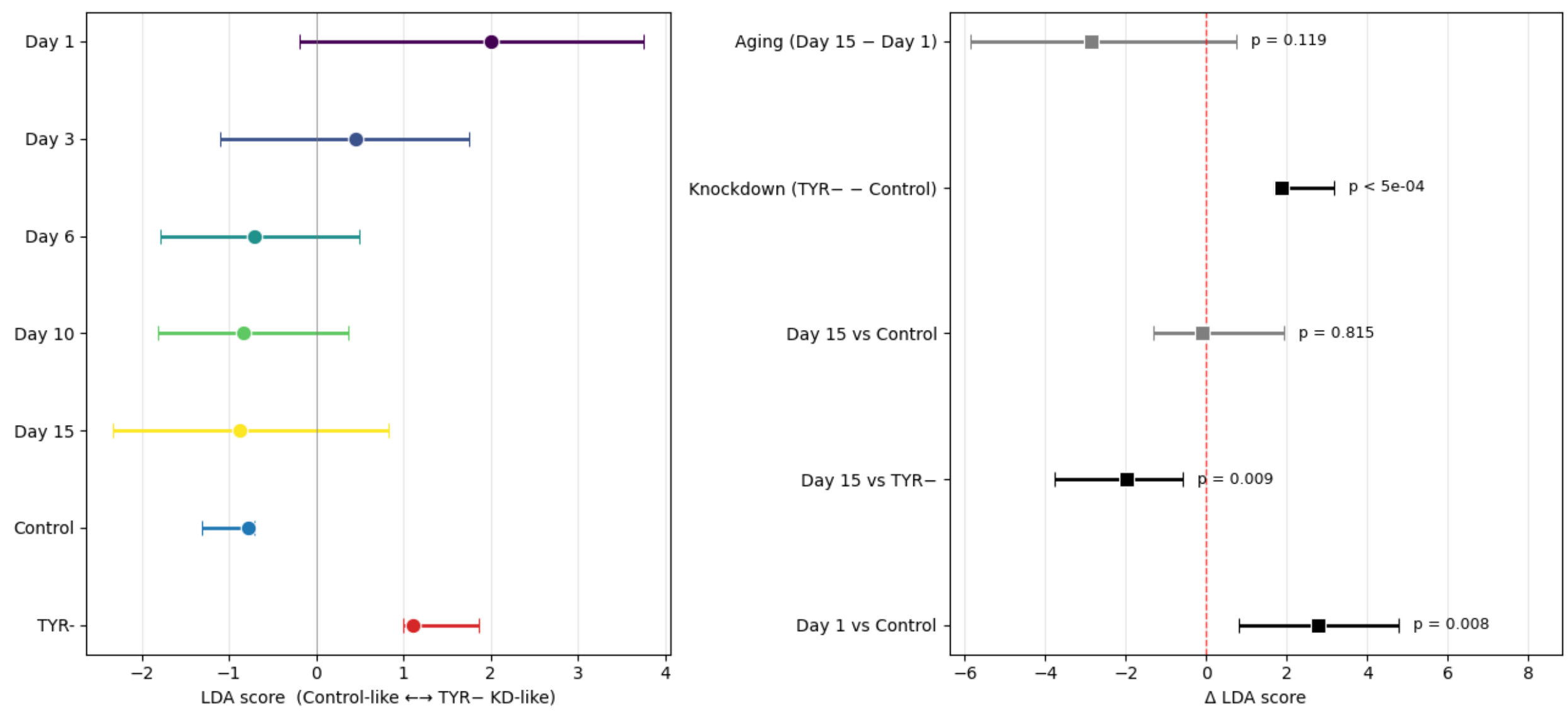

Figure S6. Left: Per-group mean LDA score (95% bootstrap CI, 2,000 boots). Right: Differences in LDA scores (95% bootstrap CI, 2,000 boots).

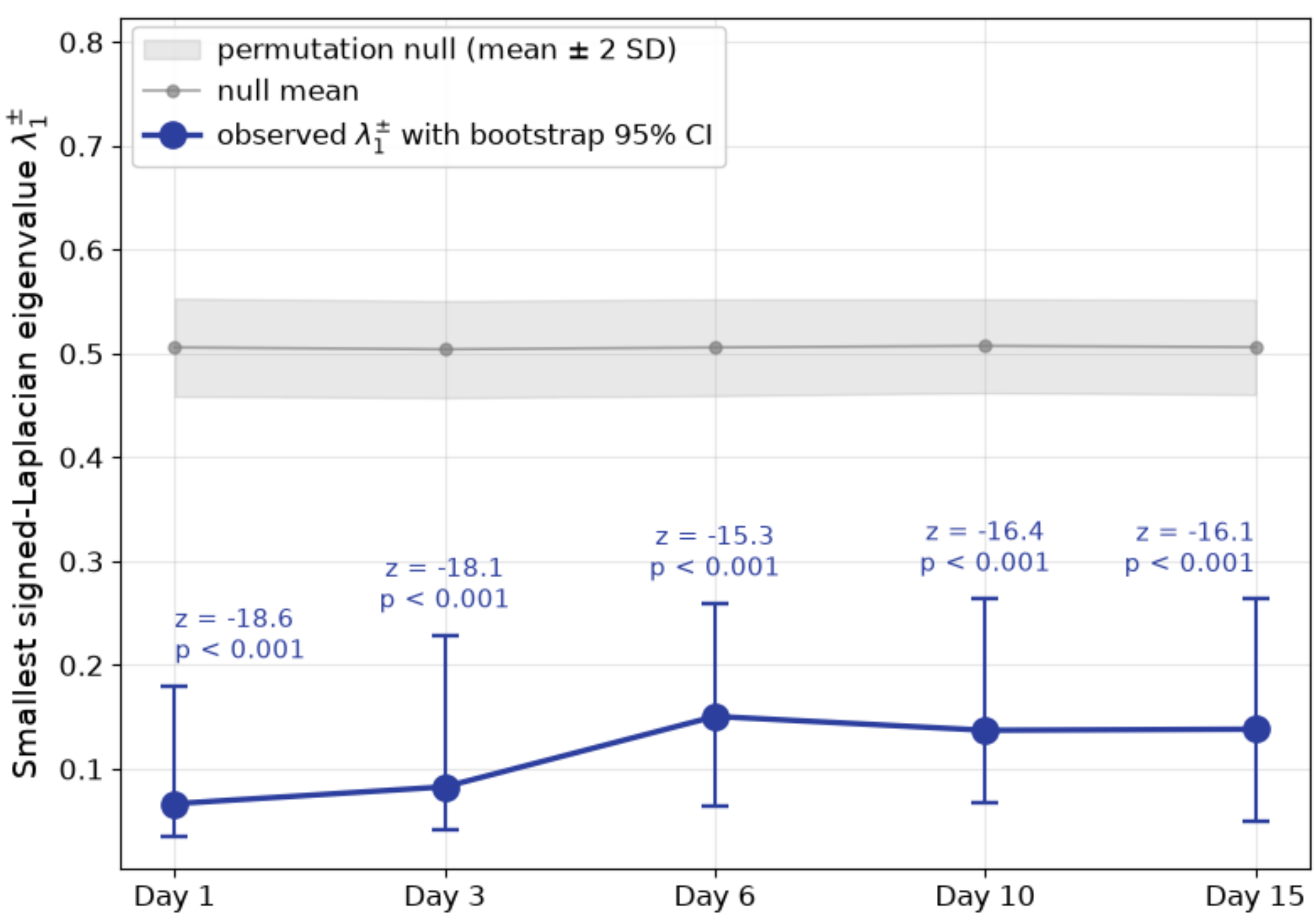

Figure S7. Observed first eigenvalue of the signed Laplacian $\lambda_1^{\pm}$, consistently below the null value.

Supplementary tables

Table S1. Genes were grouped into functional modules reflecting endocrine regulation, nutritional physiology, neural signaling, immunity, regulatory plasticity, and pollen-foraging quantitative trait loci with limited functional annotation. Functional modules were defined *a priori* based on shared biological roles and conserved signaling pathways identified from Gene Ontology annotations, pathway databases, and published functional studies in honey bees and other insects, rather than from expression-based clustering.

Module Name	Biological Description	Representative Genes
Nutritional State & Vitellogenin	Integrates nutritional status with reproductive physiology, lifespan, and division of labor via vitellogenin and insulin/TOR signaling.	vg, vgR, ILP-2, ilp1, InR1, InR2, IRS, PIK3, P110, PDK1, AKT1, PTEN, TOR, Rheb, FOXO, 4ebp1, Hex70a, Hex110, Transferrin1
Juvenile Hormone Signaling & Behavioral Maturation	Regulates age-dependent behavioral maturation through juvenile hormone transport, degradation, and transcriptional response.	JHBP-1, JHE, JHEH, Kr-h1, HR38, USP, Br-c
Ecdysteroid & Nuclear Receptor Signaling	Mediates steroid hormone–driven transcriptional programs coordinating developmental and behavioral state transitions.	EcR, E74, E75, Ftz-f1, HR46, svp
Brood Care & Secretory Physiology	Supports brood food production and nurse-specific glandular activity, including royal jelly synthesis.	MRJP1, MRJP-3, MRJP-5, MRJP-9, Mtp, Hex70a, Hex110
Neural Signaling & Foraging Behavior	Controls motivation, reward sensitivity, and foraging behavior through biogenic amine signaling and neuromodulation.	Dop1, Dop3, DopR2, OA1, Tdc1, Tdc2, Tbh, PKG, Malvolio
Immune Function & Social Physiology	Encodes innate immune responses that covary with nutritional state and social role.	Def, Def2, Hymenoptaecin, proPO
Epigenetic & Post-Transcriptional Regulation	Provides long-term transcriptional and post-transcriptional regulation	dnmt1a, dnmt2, dnmt3, AGO1, AGO2, Ago3, Aub, Dcr1, Vas, SmG

Module Name	Biological Description	Representative Genes
	underlying behavioral and physiological plasticity.	
Predicted Genes with Limited Functional Annotation	Genes within pollen-foraging QTLs with limited current functional annotation.	LOC100577942, LOC102655054, LOC406144, LOC409966, LOC410022, LOC412541, LOC413141, LOC413670, LOC724829, LOC726648, LOC726781, LOC726899

References

1. Law CW, Chen Y, Shi W, Smyth GK (2014) Voom: Precision weights unlock linear model analysis tools for RNA-seq read counts. Genome Biol 15:R29.

2. Conesa A, et al. (2016) A survey of best practices for RNA-seq data analysis. Genome Biol 17:13.

3. Daniels BC, Wang Y, Page RE Jr, Amdam GV (2023) Identifying a developmental transition in honey bees using gene expression data. PLoS Comput Biol 19:e1010704.

4. Kass RE, Raftery AE (1995) Bayes factors. J Am Stat Assoc 90:773–795.

5. Virtanen P, et al. (2020) SciPy 1.0: Fundamental algorithms for scientific computing in Python. Nat Methods 17:261–272.

6. Gómez S, Jensen P, Arenas A (2009) Analysis of community structure in networks of correlated data. Phys Rev E 80:016114.

7. Newman MEJ (2004) Fast algorithm for detecting community structure in networks. Phys Rev E 69:066133.

8. Good BH, de Montjoye YA, Clauset A (2010) Performance of modularity maximization in practical contexts. Phys Rev E 81:046106.

9. Kunegis J, Schmidt S, Lommatzsch A, Lerner J, De Luca EW, Albayrak S (2010) Spectral analysis of signed graphs for clustering, prediction and visualization. In Proceedings of the SIAM International Conference on Data Mining, pp 559–570.

10. Gallier J (2016) Spectral theory of unsigned and signed graphs: Applications to graph clustering. arXiv:1601.04692.

11. Nagel M, et al. (2020) The gene expression network regulating queen brain remodeling after insemination and its parallel use in ants with reproductive workers. Sci Adv 6:eaaz5772.

12. von Luxburg U (2007) A tutorial on spectral clustering. Stat Comput 17:395–416.

13. Ward JH Jr (1963) Hierarchical grouping to optimize an objective function. J Am Stat Assoc 58:236–244.

14. Zhang B, Horvath S (2005) A general framework for weighted gene co-expression network analysis. Stat Appl Genet Mol Biol 4:Article 17.

15. Monti S, Tamayo P, Mesirov J, Golub T (2003) Consensus clustering: A resampling-based method for class discovery and visualization of gene expression microarray data. Mach Learn 52:91–118.

16. Hennig C (2007) Cluster-wise assessment of cluster stability. Comput Stat Data Anal 52:258–271.

17. Lancichinetti A, Fortunato S (2012) Consensus clustering in complex networks. Sci Rep 2:336.

18. Hubert L, Arabie P (1985) Comparing partitions. J Classif 2:193–218.

19. Liu Z-P (2013) Linear discriminant analysis. In Encyclopedia of Systems Biology, eds Dubitzky W, Wolkenhauer O, Cho K-H, Yokota H (Springer, New York), pp 1243–1244.
